# Internalization mechanisms of brain-derived tau oligomers from patients with Alzheimer’s disease, progressive supranuclear palsy and dementia with Lewy bodies

**DOI:** 10.1038/s41419-020-2503-3

**Published:** 2020-05-04

**Authors:** Nicha Puangmalai, Nemil Bhatt, Mauro Montalbano, Urmi Sengupta, Sagar Gaikwad, Frank Ventura, Salome McAllen, Anna Ellsworth, Stephanie Garcia, Rakez Kayed

**Affiliations:** 1 Mitchell Center for Neurodegenerative Diseases, University of Texas Medical Branch, Galveston, TX 77555 USA; 2 Departments of Neurology, Neuroscience and Cell Biology, University of Texas Medical Branch, Galveston, TX 77555 USA

**Keywords:** Cellular neuroscience, Diseases of the nervous system

## Abstract

Tau aggregates propagate in brain cells and transmit to neighboring cells as well as anatomically connected brain regions by prion-like mechanisms. Soluble tau aggregates (tau oligomers) are the most toxic species that initiate neurodegeneration in tauopathies, such as Alzheimer’s disease (AD), progressive supranuclear palsy (PSP), and dementia with Lewy bodies (DLB). Exogenous tau aggregates have been shown to be internalized by brain cells; however, the precise cellular and molecular mechanisms that underlie the internalization of tau oligomers (TauO) remain elusive. Using brain-derived tau oligomers (BDTOs) from AD, PSP, and DLB patients, we investigated neuronal internalization mechanisms of BDTOs, including the heparan sulfate proteoglycan (HSPG)-mediated pathway, clathrin-mediated pathway, and caveolae-mediated pathway. Here, we demonstrated that the HSPG-mediated pathway regulates internalization of BDTOs from AD and DLB, while HSPG-mediated and other alternative pathways are involved in the internalization of PSP-derived tau oligomers. HSPG antagonism significantly reduced the internalization of TauO, prevented tau translocation to the endosomal–lysosomal system, and decreased levels of hyperphosphorylated tau in neurons, the well-known contributor for neurofibrillary tangles (NFT) accumulation, degeneration of neurons, and cognitive decline. Furthermore, siRNA-mediated silencing of heparan sulfate (HS)-synthesizing enzyme, exostosin-2, leads to decreased internalization of BDTOs, prevented tau-induced autophagy–lysosomal pathway impairment, and decreased hyperphosphorylated tau levels. Collectively, these findings suggest that HSPG-mediated endocytosis and exostsin-2 are involved in neuronal internalization of TauO and subsequent tau-dependent neuropathology in AD and DLB.

## Introduction

The formation of intracellular, hyperphosphorylated tau known as neurofibrillary tangles (NFTs) is the hallmark of several forms of dementia, including Alzheimer’s disease (AD), frontotemporal lobe degeneration (FTLD), and other tauopathies. The spatial and temporal appearance of NFT correlates with cognitive decline and clinical severity of symptoms in AD patients^1^. Various studies have described mechanisms of tau protein transmission from neuron-to-neuron and between anatomically connected brain regions via a trans-synaptic mechanism^2,3^. Despite these evidences, the mechanism of BDTOs internalization in neurons and subsequent tau-dependent neuropathology remains unclear. Previous findings suggest that tau forms distinct strains that propagate and stably maintain the unique conformations in vitro and in vivo. In addition, tau targets different brain regions and propagates pathology at unique rates^4^; however, the strain-specific release and internalization of tau is not well understood.

Heparan sulfate proteoglycans (HSPGs) are ubiquitous cell surface molecules composed of a core protein conjugated with multiple copies of heparan sulfate (HS), a linear polysaccharide. HSPGs are present on the surface of almost all types of mammalian cells, which are involved in processes of cell development, differentiation, attachment, and inflammation^5^. Several lines of evidence suggest that HSPGs can interact with protein aggregates, including tau, amyloid-β, and α-synuclein^6–9^. We speculate that the HSPG-mediated endocytosis pathway could be involved in internalization of BDTOs. Exostosin-2 (Ext2), an HS-synthesizing enzyme, belongs to the exostosin family, which catalyzes polymerization of glucuronic acid and N-acetylglucosamine^10^. This enzyme has been involved in axonal growth, as well as neuronal injury^11^, and plays a role as a tau uptake modulator in human cell lines^12,13^. Although the role of HSPG in tau internalization has been extensively studied, the internalization mechanisms of distinct BDTOs from human tauopathies are still undefined. In this study, using primary cortical neurons, we examined the involvement of HSPG-mediated, clathrin-mediated, and caveolae-mediated pathways during the internalization of BDTOs from AD, PSP, and DLB brain tissues. We found that HSPG plays a role in the primary internalization pathway for AD and DLB tau oligomers, while HSPG-mediated and other alternative pathways are utilized for PSP tau oligomers uptake in neurons. Exogenous TauO caused alterations of the autophagy–lysosomal pathways (ALP) and enhanced levels of phosphorylated tau after internalization, which were attenuated by Exostosin-2 knockdown. These findings implicate targeting the HSPG-mediated endocytosis pathway and exostosin-2 could be helpful to decrease/prevent tau-dependent pathologies such as AD and DLB.

## Materials and methods

### Brain homogenate preparation

Postmortem brain tissue samples from AD, PSP, and DLB were obtained from Oregon Health and Science University, the Institute for Brain Aging and Dementia (University of California–Irvine, Irvine, CA, USA) and the Brain Resource Center at Johns Hopkins. Neuropathological assessment conformed to National Institute on Aging/Reagan Institute consensus criteria. The following information was available for the cases used in this study: diagnosis, age at death, gender, postmortem index, brain area, and Braak stage (Supplementary Table S1). Each brain was homogenized in PBS with protease inhibitor cocktail (Roche; 11836145001), using 1:3 dilution of brain: PBS (w/v). Samples were centrifuged at 10,000 rpm for 10 min at 4 °C. Supernatants were aliquoted, snap-frozen, and stored at −80 °C until use.

### Immunoprecipitation of tau oligomers from human brain tissues

Immunoprecipitation (IP) experiments were performed as previously described^14^. Briefly, tosyl-activated magnetic Dynabeads (Dynal Biotech, Lafayette Hill, PA) were coated with 20 μg of T22 antibody (1.0 mg/ml) diluted in 0.1 M borate, pH 9.5, overnight at 37 °C. Beads were washed (0.2 M Tris, 0.1% bovine serum albumin, pH 8.5) and then incubated with either AD, PSP, or DLB brain homogenate (PBS soluble fraction) with rotation at room temperature for 1 h. Beads were then washed three times with PBS and eluted using 0.1 M glycine, pH 2.8. The pH of each eluted fraction was adjusted using 1 M Tris pH 8.0. Fractions were then centrifuged in a microcon centrifugal filter device with a molecular weight cutoff of 10 kDa (Millipore; 42415) at 14,000 × *g* for 25 min at 4 °C. Oligomers were then resuspended in sterile PBS. Total protein concentration was determined using the bicinchoninic acid protein assay (Pierce). The samples were again centrifuged in a microcon centrifugal filter device with a cutoff of 10 kDa at 14,000 × *g* for 25 min at 4 °C. Oligomers were then resuspended in PBS in order to obtain the desired concentration (0.1–0.5 mg/ml), and kept at −20 °C. No oligomers were found in the IP from control brains, as previously reported by our group^15^ and others^16^. The characterization and seeding assay of BDTOs^17^ from AD^15,18^, PSP^19^, and DLB^20^ brains were previously published from our laboratory and explained in more detail in Supplementary information and Supplementary Fig. S1. All BDTOs were detected the prion-like activity in tau RD P301S biosensor cells. More details are explained in Supplementary information and Supplementary Fig. S2.

### Fluorescent and biotin labeling of tau protein

BDTOs and fibrils were labeled with Alexa Fluor™ (AF568 or AF488) NHS Ester (Invitrogen) according to the manufacturer’s guideline with minor modifications. Briefly, AF568 or AF488 NHS Ester was dissolved in 100 mM sodium bicarbonate to make the final concentration 1 mg/ml. The dye solution was then incubated with TauO in a 1:2 ratio (w/w). The mixture was rotated overnight at 4 °C. The following day, the solution was centrifuged at 15,000 × *g* for 30 min using 10 kDa Amicon Ultra-0.5 Centrifugal Filter Units to remove unbound dye. Oligomers were subsequently washed with PBS. Filter compartment was centrifuged to collect the concentrate.

For tau biotinylation, EZ-Link NHS-PEG4-Biotin, No-Weigh Format (2 mg, Thermo Scientific; A39259) was reconstituted in water to create a 2 mM stock solution. TauO was incubated with the biotin reconstituted stock at a 1:1 molar ratio for 30 min at room temperature (RT) or 2 h on ice. The biotinylated protein was then purified using Zeba desalting spin columns (Thermo Scientific; 89882) according to the manufacturer’s instructions.

### Primary neuron isolation and cell treatment

This study was conducted in a facility approved by the American Association for the Accreditation of Laboratory Animal Care. All procedures were performed in accordance with recommendations in the Guide for the Care and Use of Laboratory Animals of the National Institutes of Health. Our protocol was approved by the Institutional Animal Care and Use Committee of the University of Texas Medical Branch (UTMB). Primary cortical neuronal cultures were prepared and maintained as described previously^21^. Briefly, cortical neurons were isolated from C57BL/6 mice (Jackson Laboratory; 000664) during embryonic days 16–18 using Accutase solution (Sigma; A6964) together with gentle trituration by a fire-polished glass pasture pipet. Dissociated cells were plated at a density of 2 × 10^5^ cells/ml in a 24-well plate containing high glucose Dulbecco’s Modified Eagle’s Medium (DMEM, Corning; 10–013-CV) with 2% B-27 Plus supplement (Gibco; A3582801), 10,000 U/ml penicillin, 10,000 μg/ml streptomycin, and 25 μg/ml amphotericin B (Gibco; 15240062). After 2 h, plating medium was removed from cells and replenished with neurobasal medium (Gibco; 12348017) plus 2% B-27 Plus, 0.5 mM GlutaMax (Gibco; 35050-061), 10,000 U/ml penicillin, 10,000 μg/ml streptomycin, and 25 μg/ml amphotericin B supplement. In all, 50% of medium changes were performed every 3–5 days. Cells on days 10–13 in vitro (DIV) were used for all experiments.

For cell treatment, neurons cultured in a 24-well plate were exposed to pharmacological inhibitors for 30 min, being either dynamin inhibitor (Dynasore (Sigma, D7693) or Pitstop2 (Abcam, ab120687)), HSPG inhibitor (Heparin (Sigma, H4784)), or caveolae inhibitor (Nystatin (Sigma, N6261)). AF568- or AF488-tagged TauO exposure were further performed for 1–24 h. Concentrations of inhibitors and TauO and incubation time were mentioned in appropriate methods and figure legends. DMSO (0.02% (v/v)) was the vehicle control in all experiments.

### Tau internalization assay using flow cytometry

Cells cultured in a 24-well plate were 30 min exposed with either Dynasore or PitStop2 (26–38 μg/ml), Heparin (1–200 μg/ml), or nystatin (10–40 μg/ml). After several washes, AF568-tagged TauO at a concentration of 0.5 μM (or equivalent protein concentration at 24 μg/ml) were further incubated. After 1 h incubation, cells were washed once with Hank’s balanced salt solution (Corning) followed by trypsinization with Accutase solution at 37 °C for 10 min to quench surface-bound fluorescence. Cell pellets were collected via centrifugation at 300 × *g* for 10 min, and resuspended in flow cytometry buffer (HBSS with 1% fetal bovine serum (Gibco) and 1 mM EDTA (Sigma)). To quantify tau internalization, cells were counted to 10,000 cells, gated for viable and singlet cells, and further determined the median fluorescence intensity (MFI) per cell (shown as histogram) using LSRII Fortessa Analyser (BD Biosciences). The percentage of AF568-tagged tau-positive cells of each experiment was combined for bar graphs. Each experiment was conducted three times with technical triplicates. Data analysis was performed using FlowJo v10 software (Treestar Inc.) and Prism 6.0 (GraphPad).

### Immunostaining, confocal microscopy, and imaging analysis

Cells were grown on poly-L-Lysine-coated coverslips. After TauO application for the indicated time, 4% formaldehyde (Sigma) was used for fixation for 15 min at RT followed by three washes with PBS. Cells were permeabilized using 0.25% Triton X-100 in PBS for 10 min and blocked for 30 min in 5% goat serum (Sigma) at RT. After blocking, cells were incubated with primary antibodies diluted in blocking buffer overnight at 4 °C. Primary antibodies used include: mouse anti-βIII-tubulin (1:1000, Abcam; ab78078), rabbit anti-Rab5 (1:500, Abcam; ab13253), and rabbit anti-LAMP-2 (1:500, Invitrogen; PA1-655). On the next day, cells were washed and incubated with secondary antibodies (1:1000, Life Technologies) for 1 h at RT. After three washes, cells were mounted with ProLong Diamond antifade mounting media with or without DAPI (Invitrogen; P36971, P36970). All samples were examined with 63x objective of a Zeiss LSM 880 confocal microscope using 405-nm diode laser and argon laser 458/488/514 nm. To build the z-stack, 17 stacks/0.37–0.41-µm optimal thickness were captured. Each treatment condition was randomly imaged in five different regions of interest and performed in duplicate. All images were analyzed via Pearson’s Correlation Coefficient for cellular compartment colocalization with TauO using ImageJ (NIH).

### siRNA transfection

Silencing gene of interest was carried out using Accell SMARTpool siRNA (Dharmacon), which contains four siRNAs of usage according to the manufacturer’s directions. Briefly, 48 h after plating cells, neurons were transfected with 500 nM siRNA that was diluted in neurobasal medium. Cells were maintained in siRNA-containing medium for 96 h. The efficiency of the gene knockdown was evaluated using real-time quantitative polymerase chain reaction (RT-qPCR) and Western blotting. Non-targeting (NT) siRNAs were used as control. All target sequences are shown in Supplementary Table S2.

### RNA isolation and RT-qPCR

The total RNAs from siRNA-treated neurons were extracted using TRIzol reagent following the established protocol. RNA samples for real-time analysis were quantified using a Nanodrop Spectrophotometer (Nanodrop Technologies), and qualified by analysis on a RNA Nano chip using the Agilent 2100 Bioanalyzer (Agilent Technologies). Samples with high-quality total RNA were used (RIN: 7.5–10.0). Synthesis of cDNA was performed with 0.5–1 µg of the total RNA in a 20-µl reaction using the reagents in the Taqman Reverse Transcription Reagents Kit from Life Technologies (N8080234). Q-PCR amplifications were done using 1 µl of cDNA in a total volume of 20 µl using the iTaq Universal SYBR Green Supermix (Bio-Rad; 1725125). The final concentration of the primers was 300 nM. Relative RT-qPCR assays were performed with 18S RNA housekeeping gene as a normalizer. Relative analysis was performed using known amounts of a synthetic transcript of the gene of interest. All PCR assays were run in the ABI Prism 7500 Sequence Detection System. A list of primers used is available on Supplementary Table S3.

### Cell lysate collection

Cells (5 × 10^5^ cells/ml) were cultured in a six-well plate. After 18 h treatment of TauO with or without siRNA pre-exposure, total cell lysates were collected using 1× RIPA buffer (Cell Signaling; 9806) supplemented with 2% protease/phosphatase inhibitor (Roche; 04906837001). Cells were scraped, resuspended in ice-cold lysis buffer, and centrifuged at 13,000 × *g* for 10 min. Supernatant was collected and quantified the total protein using Pierce^TM^ BCA protein assay kit (Thermo Scientific; 23225).

### Western blot analysis

Pre-cast NuPAGE 4–12% Bis-Tris Gels for SDS-PAGE (Invitrogen) were loaded with 10–20 μg/well of cell lysate, ran under reducing conditions, and transferred to nitrocellulose membranes. After blocking for 1 h at RT with 10% nonfat dried milk, membranes were incubated with mouse anti-EXT2 (1:1000, Santa Cruz Biotechnology; sc-514092), mouse anti-p62/SQSTM1 (1:1000, Abcam; ab56416), rabbit anti-LC3B (1:1000, Novus Biologicals; NB100-2220), rabbit anti-LAMP-2 (1:1000, Invitrogen; PA1-655), mouse anti-p-Tau (Thr231) (AT180) (1:1000, Thermo Scientific; MN1040), mouse anti-p-Tau (Ser202, Thr205) (AT8) (1:1000, Thermo Scientific; MN1020), Streptavidin-HRP (Southern Biotech; 7100-05), mouse anti-Tau-5 (1:15000, Bio Legend; 806402), rabbit anti-Tau (1:2000, Abcam; ab64193) for total tau, mouse anti-βIII-tubulin (1:1000, Abcam; ab78078) as loading control. Primary antibodies were diluted in 5% nonfat dried milk overnight at 4 °C. Immunoreactivity was detected with horseradish peroxidase-conjugated IgG anti-rabbit and anti-mouse secondary antibody, respectively (1:10,000, GE Healthcare). For signal detection, ECL plus (GE Healthcare) was used. Densitometry of each band was quantified and normalized with internal control using ImageJ (NIH).

### Statistical analysis

The number of replica wells and experiments were indicated in the figure legends for each assay when appropriate. Imaging analysis is previously described in “Immunostaining, confocal microscopy, and imaging analysis”. One-way analysis of variance (ANOVA) was performed using Prism 6.0 (GraphPad Software, Inc., San Diego, CA, USA) to measure statistical significance of differences, followed by Tukey’s test. The unpaired and two-tailed Student’s *t* test was used when appropriate. Data are presented as the mean ± SEM of BDTOs from three different brains in triplicate. The results are considered statistically significant at *p* < 0.05.

## Results

### HSPG antagonist inhibits brain-derived tau oligomers internalization and cytotoxicity in neurons

**Fig. 1:**
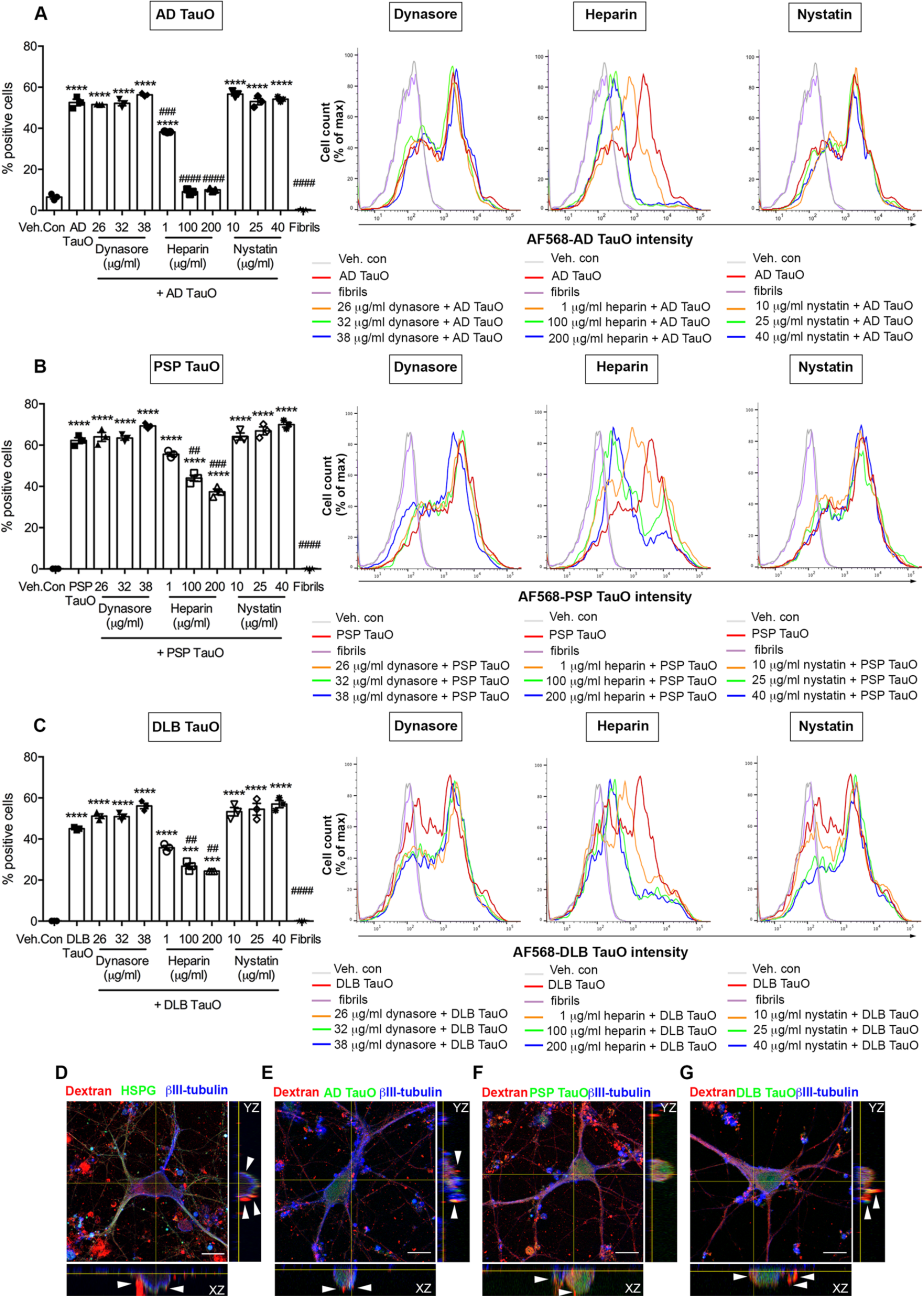
HSPG antagonist reduces BDTOs internalization in neurons. **a**–**c** Primary cortical neurons were pretreated with internalization inhibitors for 30 min before treating with 0.5 μM BDTOs for 1 h. AD TauO (**a**), PSP TauO (**b**), and DLB TauO (**c**) internalized into cells ~55%, 60%, and 45%, respectively. The percentage of AF568-tagged tau-positive cells and median fluorescence intensity (right panel) from AD TauO (**a**), PSP TauO (**b**), and DLB TauO (**c**) applications were significantly diminished in a concentration-dependent fashion by Heparin (a HSPG antagonist), whereas Dynasore (a clathrin inhibitor) and Nystatin (a caveolae inhibitor) did not show inhibitory effects. A total of 10,000 cells were analyzed for each condition in triplicate. Error bars show SEM. ***p < 0.001, ****p < 0.0001 vs vehicle control (Veh. Con; 0.02% (v/v) DMSO), ^##^*p* < 0.01, ^###^*p* < 0.001, ^####^*p* < 0.0001 vs TauO-treated. All histograms (right panel) represent integrated median fluorescence intensity of BDTOs in cells pretreated with 26–38 μg/ml Dynasore, 1–200 μg/ml Heparin, or 1–40 μg/ml Nystatin. AF568-tagged tau fibrils were used as the negative control in all experiments. See also Supplementary Fig. S3a. **d**–**g** Dextran and BDTOs uptake were associated with HSPG. A total of 20 μg/ml of 70,000 MW lysine-flexible Texas Red-conjugated dextran was applied to cells for 1 h. **d** Cells were fixed and immunostained for HSPG (green) and a mature neuronal marker (βIII-tubulin, blue). Representative orthogonal images indicated AF488-tagged AD TauO (**e**), PSP TauO (**f**), and DLB TauO (**g**) co-localized to dextran, indicating the HSPG-mediated uptake. Scale bar: 10 μm.

Exogenously applied tau protein has been shown to be internalized by neurons and other cell types^22–24^. We investigated the involvement of HSPG-mediated endocytosis pathway, clathrin-mediated pathway, and caveolae-mediated pathway during neuronal internalization of TauO from AD, PSP, and DLB. We first analyzed the internalization mechanisms of tau oligomers. We observed the ability of distinct brain-derived TauO from AD, PSP, and DLB to enter primary cortical neurons after extracellular application when compared with tau fibrils. To test the specific internalization mechanisms in neurons for each TauO, we used selective pharmacological inhibitors of the major physiological endocytosis pathways. This includes inhibitors for clathrin-mediated (Dynasore or PitStop2), HSPG-mediated (Heparin), and caveolae-mediated (nystatin) endocytosis. To determine the efficacy of different BDTOs internalization in primary neurons, we labeled BDTOs or tau fibrils with AF568 protein-labeling dye for fluorescence detection. Cells were initially cultured in the presence or absence of internalization inhibitors for 30 min. After the culture medium removal, cells were further washed and incubated with a sublethal concentration of 0.5 μM AF568-tagged TauO or fibrils for 1 h. Optimal concentration and time were determined empirically. To remove the cell membrane-bound tau, cells were repeatedly washed and collected using Accutase prior to flow cytometry analysis. We found that different BDTOs had diverse internalization efficiencies. Internalized AD TauO (Fig. 1a) was detected in almost 55% of cell population, whereas PSP TauO (Fig. 1b) and DLB TauO-positive cells (Fig. 1c) were detected in 60% and 45% of total cell population (*p* < 0.0001), respectively. In contrast, Heparin pretreatment showed significant reduction in TauO internalization in a dose-dependent manner for AD TauO, PSP TauO, and DLB TauO-positive cells (*p* < 0.0001), which was not observed in Dynasore (*p* = 0.9968) (or PitStop2; data not shown) nor Nystatin (*p* = 0.9940) pretreated groups. Representative histograms (right panels) showed a notable reduction of fluorescence intensities of all the examined BDTOs when pre-exposed to high concentrations (100–200 μg/ml) of Heparin. However, Dynasore and Nystatin did not inhibit tau internalization at 1-h incubation timepoint. Flow cytometry analysis revealed that tau fibrils were not internalized by neurons, but instead they bound to the cell membrane (wheat germ agglutinin-positive) illustrated by immunofluorescence staining (Supplementary Fig. S3a). Further, to confirm the HSPG-dependent neuronal internalization pathway, we examined the colocalization of HSPG with internalized dextran (Molecular Probes; D1864)^25^. The orthogonal image showed colocalization of dextran with HSPG (Fig. 1d). Colocalization of AF488-tagged AD TauO (Fig. 1e), PSP TauO (Fig. 1f), and DLB TauO (Fig. 1g) with dextran, shown in orthogonal views, confirmed that the internalization pathway is HSPG-dependent.

Since prion-like propagation of tau seeds is involved in progressive neurodegenerative diseases such as tauopathies, we investigated role of HSPG on the prion-like activity of BDTOs. Tau RD P301S biosensor cells were used to detect the seeding activity in the present or absent of Heparin after 24 h of treatment. We found that BDTOs/Heparin mixture showed drastic reduction of seeding activity (Supplementary Fig. S6a) compared with BDTOs-treated group. Quantification of FRET-positive cells represented significant reduction of seeding activity observed on the three different BDTOs in the presence of Heparin (Supplementary Fig. S6b–d).

Many studies suggest that oligomeric tau potentiates neuronal damage, leading to neurodegeneration^15,19,26^. To further identify the effect of the HSPG antagonist on TauO-induced toxicity in neurons over 24 h, LDH release was detected from culture media after cells were treated with TauO in the presence or absence of Heparin pretreatment. Heparin (200 μg/ml) significantly showed a protective effect against AD, PSP, and DLB TauO-induced LDH leakage (*p* < 0.0001) (Supplementary Fig. S3b). These results suggest that HSPG-mediated endocytosis is involved in the internalization of brain-derived tau oligomers, and HSPG antagonism prevents TauO-mediated proteopathic tau seeding and neurotoxicity.

### Internalized brain-derived tau oligomers bind to postsynaptic marker and localize with endosomal–lysosomal system

**Fig. 2:**
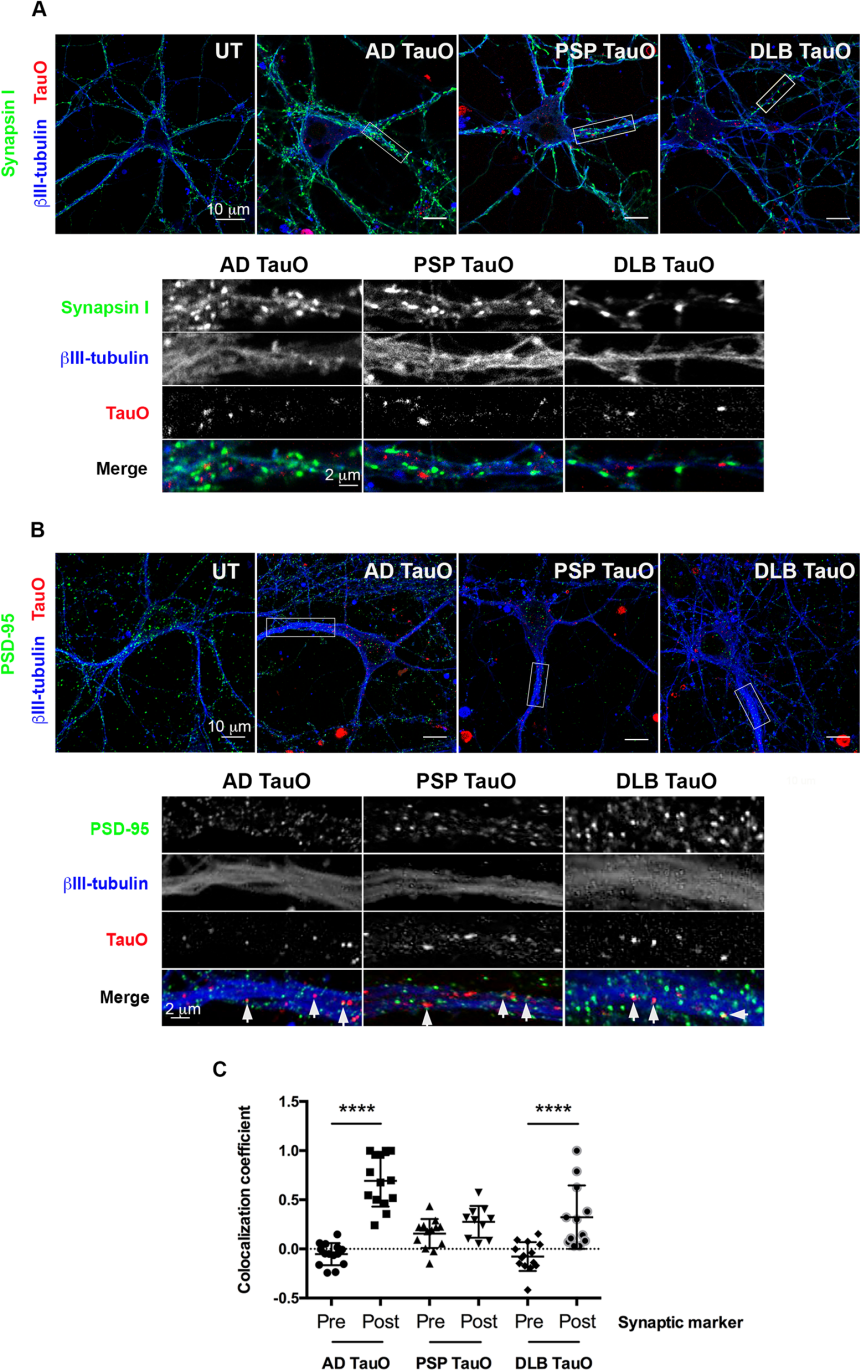
Exogenous tau oligomers bind to postsynaptic marker. Neurons were exposed to AF568-tagged TauO (Red) from AD, PSP, or DLB for 1 h. Cells were immunolabeled with a presynaptic marker (Synapsin I, green) (**a**), a postsynaptic marker (PSD-95, green) (**b**), and a mature neuronal marker (βIII-tubulin, blue). Representative regions of interest are depicted in white rectangles with inserted high-magnification below. Scale bar is indicated. AD, PSP, or DLB TauO are located near the presynaptic marker. White arrows indicate AD, PSP, or DLB TauO co-localized with the postsynaptic marker. **c** Pearson’s correlation coefficient analysis of exogenous AD, PSP, and DLB TauO with pre- and postsynaptic markers over 1 h. Each treatment group was randomly imaged in five different regions of interest, and performed in triplicate. Image analyses were calculated by one-way ANOVA with Tukey’s multiple comparison test. Results showed as the value of mean ± SEM, ****p < 0.0001.

The trans-synaptic and trans-neuronal propagation of tau has been implicated in the progression of tau-mediated neurodegeneration^27,28^. The different TauO could bind and spread differently via synaptic proteins. To test this hypothesis, cortical neurons were exposed to AF568-tagged TauO for 1 h. Immunostaining was initially performed to investigate the localization of BDTOs with synaptic protein markers. As early as 1 h after the extracellular TauO addition, exogenous TauO were primarily observed adjacent to the presynaptic marker (Synapsin I) (Fig. 2a) and co-localized with postsynaptic marker (PSD-95) (Fig. 2b) along the neuronal processes. Analyses of colocalization coefficient (Fig. 2c) confirmed the notably increased binding of AD TauO and DLB TauO to postsynaptic marker (*p* < 0.0001), whereas PSP TauO bound to postsynaptic marker was slightly higher than presynaptic marker without reaching significance (*p* = 0.1271), suggesting that TauO from AD and DLB, but not PSP, colocalize with postsynaptic marker, PSD-95.

**Fig. 3:**
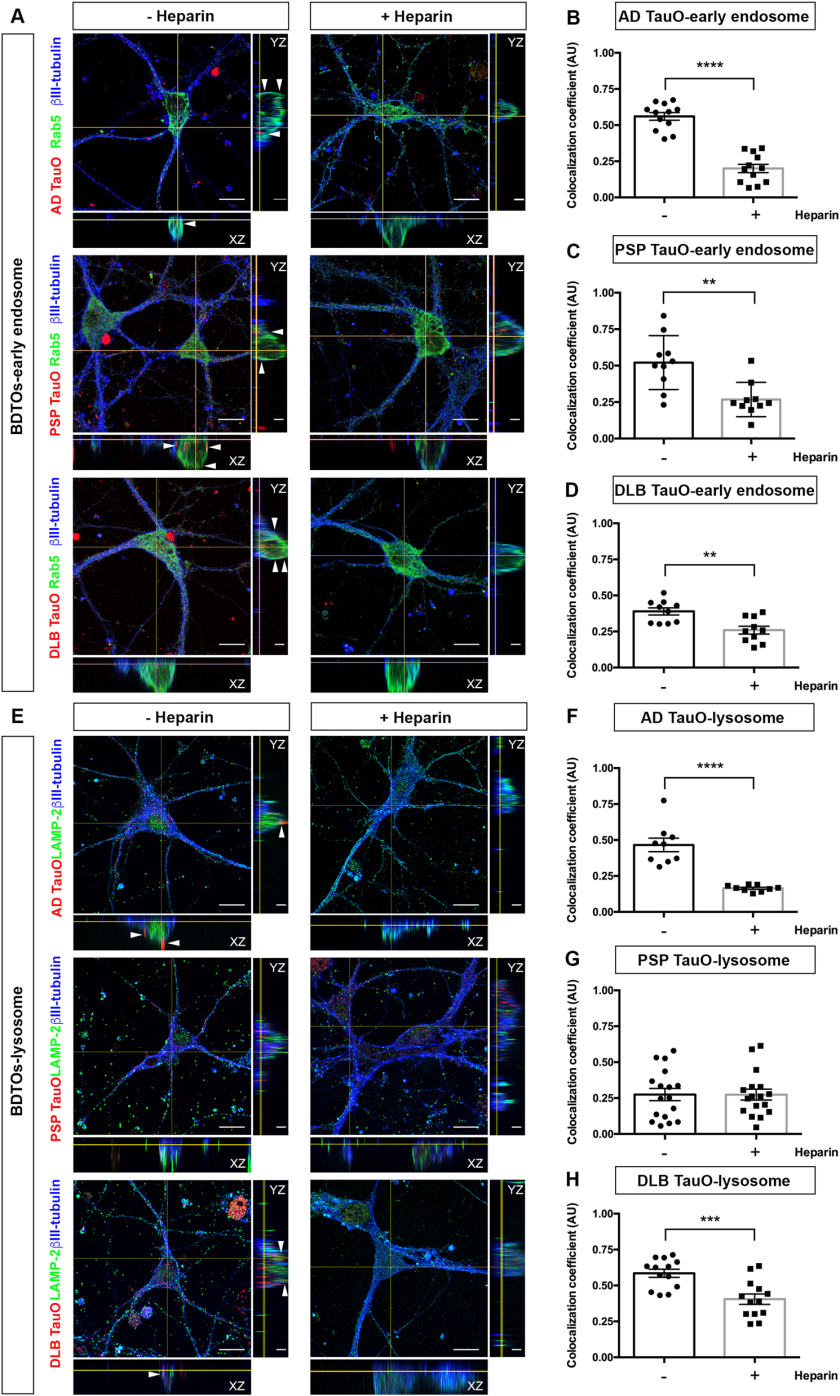
Localization of internalized tau oligomers with the endosomal– lysosomal system. **a**, **e** Neurons were exposed to AF568-tagged TauO (Red) from AD, PSP, or DLB for 1 h with (+) or without (−) Heparin pretreatment. Cells were fixed and immunostained for a mature neuronal marker (βIII-tubulin, blue), an early endosomal marker (Rab5, green) (**a**) and a lysosomal marker (LAMP-2, green) (**e**). Representative orthogonal images indicate AF568-tagged TauO co-localized to early endosomes and lysosomes, indicated by arrows. Scale bar: 2 and 10 μm. **b**–**d** Pearson’s correlation coefficient analysis of internalized AD TauO (**b**), PSP TauO (**c**), DLB TauO (**d**) with early endosome over 1 h was demonstrated with similar experimental conditions and parameters as for (**a**). Each treatment group was randomly imaged in five different regions of interest, and performed in duplicate. **f**–**h** Pearson’s correlation coefficient analysis of lysosome with internalized AD TauO (**f**), PSP TauO (**g**), and DLB TauO (**h**) was demonstrated, using the same experimental conditions as for (**e**). Image analyses were calculated by unpaired and two-tailed Student’s *t* test. Results showed as the value of mean ± SEM, **p < 0.01, ***p < 0.001, ****p < 0.0001 vs without (−) Heparin.

In mammalian cells, the endocytic pathway is initiated by the fusion of extracellular materials into early endosomes followed by either releasing of internalized cargo outside the cells or fusing with late endosomes and lysosomes for further degradation^29^. We established the route of TauO internalization and role of Heparin in the exogenous TauO translocation. We analyzed the appearance of AF568-labeled TauO in early endosomes (Rab5) and lysosomes (LAMP-2) using confocal microscopy with colocalization analysis. We found that extracellular TauO from AD, PSP, and DLB were co-localized with early endosomes (Fig. 3a–d) and lysosomes (Fig. 3e–h). In agreement with flow cytometry analysis, Heparin blocked endocytosis for AD and DLB TauO to early endosomes in cell bodies as well as in neuronal processes (Fig. 3a), which is confirmed by the colocalization coefficient values (Fig. 3b, d). In PSP TauO-exposed groups, reduction in colocalization of tau with early endosomal marker protein were noted following Heparin treatment (Fig. 3c). However, we still observed the internalized of TauO (Fig. 3a), suggesting that other mechanisms might play roles in PSP TauO uptake. Heparin administration not only demonstrated reduced AD and DLB TauO uptake in endosomes but also inhibited lysosome transfer of both TauO (Fig. 3f, h). On the contrary, Heparin had no effect on PSP TauO colocalization with lysosomes (Fig. 3g).

### Exostosin-2 regulates accumulation of brain-derived tau oligomers in neurons

**Fig. 4:**
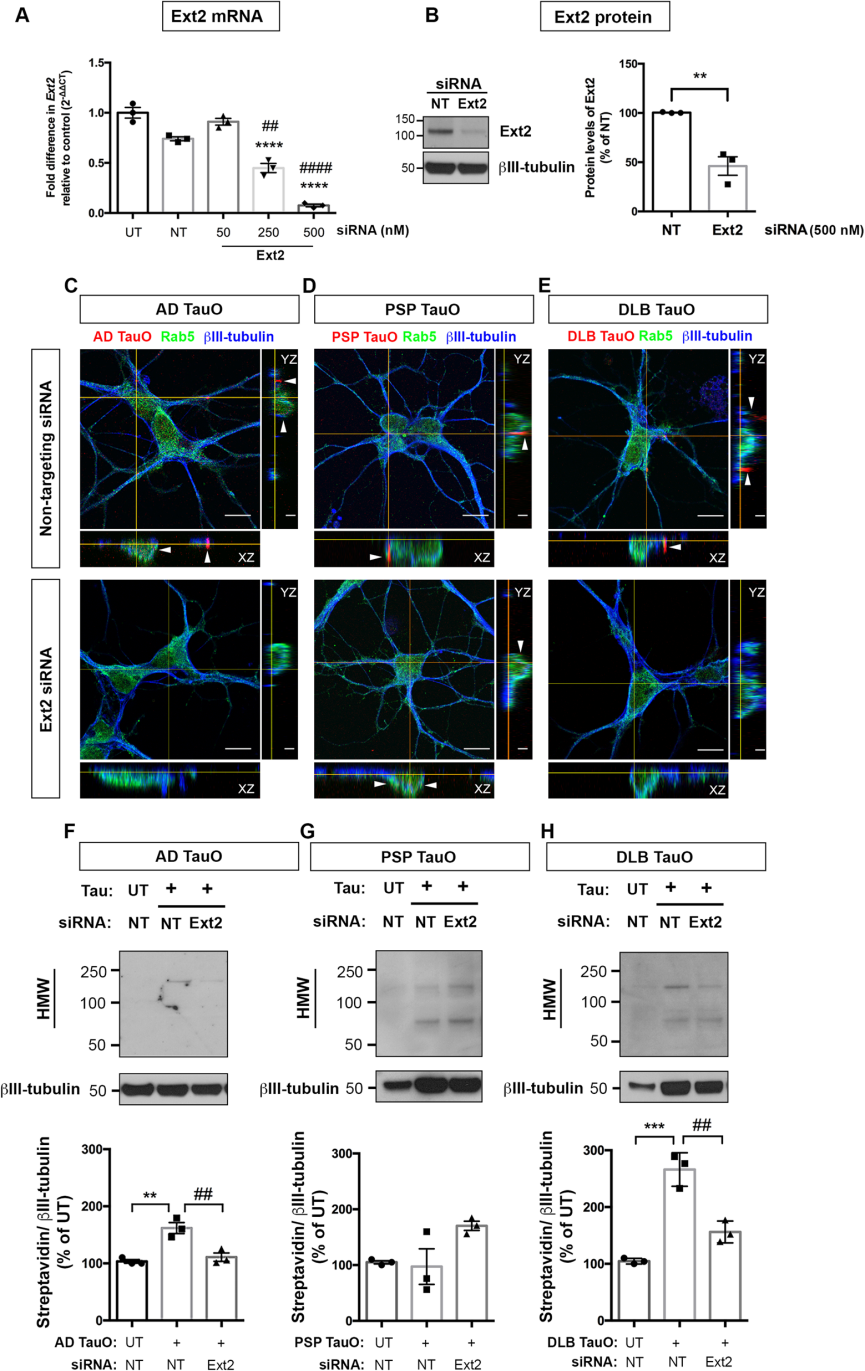
Exostosin-2 knockdown prevented internalization and accumulation of extracellular tau species in neurons. **a**, **b** Primary neurons (DIV2) were applied Accell^TM^ SMARTpool siRNA for non-targeting (NT) or Ext2 gene (NM_001355075) at concentrations of 50–500 nM for 96 h. 18s rRNA was used for the reference gene and normalization in gene expression analysis. Ext2 siRNA (500 nM) significantly reduced the exostosin-2 mRNA (**a**) and protein (**b**) levels compared with NT siRNA. Results were from three independent experiments and shown as fold difference expression relative to control (mean ± SEM). Statistical analyses were measured by one-way ANOVA with Tukey’s test (**a**) or unpaired, two-tailed Student’s *t* test (**b**) from three biological independent experiment. Results showed as the value of mean ± SEM, **a** ****p < 0.0001 vs UT group, ^##^*p* < 0.01, ^####^*p* < 0.0001 vs NT group, **b** **p < 0.01 vs NT group. **c**–**e** Neurons (DIV2) were preincubated with NT or Ext2 siRNA for 96 h followed by AF568-tagged TauO from AD (**c**), PSP (**d**), or DLB (**e**) treatment for 18 h. Cells were immunolabeled with a mature neuronal marker (βIII-tubulin, blue), and an early endosomal marker (Rab5, green). Representative orthogonal images depicted AF568-tagged TauO co-localized to early endosomes (arrows). Scale bar: 2 and 10 μm. **f**–**h** Neurons were untreated (UT) or treated with 0.1 μM biotin-tagged TauO (+) from AD (**f**), PSP (**g**), or DLB (**h**) with similar experimental conditions and parameters as for (**c**–**e**). Internalized tau was detected using anti-Streptavidin antibody. Representative Western blot images depicted the appearance of exogenously applied TauO. Internal controls from the same blot were probed with anti-βIII-tubulin. Analysis of internalized tau levels was on the lower panel of each immunoblot showing as Streptavidin band intensity (HMW: 75–250 kDa) normalized to internal control and presented as the percentage of UT group. Statistical analyses were measured by one-way ANOVA with Tukey’s test from three biological independent experiments. Results showed as the value of mean ± SEM, **p < 0.01, ***p < 0.001 vs UT group. ^##^*p* < 0.01 vs TauO-treated group. Western blot analyses of TauO from AD, PSP, or DLB were performed on separate membranes. The same membranes were re-probed for marker proteins of autophagy–lysosomal pathway as shown in Fig. 5a–c. The immunoblots for internal controls shown in Fig. 4f–h were reused in Fig. 5a–c.

To explore further whether HSPG is essential for BDTOs internalization in neurons, we performed siRNA-mediated knockdown of the enzyme for heparan sulfate (HS) biosynthesis, Ext2. First, we tested the silencing efficiency of siRNA application using RT-qPCR. At 96 h after siRNA transfection, the downregulation of *ext2* was observed in a concentration-dependent manner (Fig. 4a). The optimal Ext2 siRNA concentration (500 nM) significantly downregulated the mRNA levels relative to untreated (UT) and non-targeting (NT) siRNA control (*p* < 0.0001), as well as lessened the Ext2 protein levels to 50% compared with NT siRNA group (*p* = 0.0045) (Fig. 4b). Toxicity of siRNA application was determined by LDH levels, and no significant neuronal damage was observed (data not shown).

We analyzed the ability of Ext2 siRNA to inhibit AD, PSP, and DLB TauO internalization by neurons. Cells were treated with NT or Ext2 siRNA followed by AF568-tagged TauO (0.1 μM) for 18 h. We found that Ext2 siRNA inhibited the internalization of AD TauO (Fig. 4c) and DLB TauO (Fig. 4e), but not PSP (Fig. 4d). The results were confirmed by Western blot analyses of biotinylated TauO-treated neurons at similar timepoint. We found that Ext2 siRNA significantly reduced the uptake AD TauO (Fig. 4f) and DLB TauO (Fig. 4h) compared with NT siRNA-treated group. However, the ext-2 knockdown did not inhibit the PSP TauO internalization (Fig. 4g). These results indicated that HSPG-mediated neuronal uptake primarily plays a role in AD and DLB TauO internalization, however, other mechanisms may take part in PSP TauO neuronal uptake.

### Knockdown of Ext2 attenuated exogenous tau oligomers disturbed the autophagy–lysosomal pathway (ALP)

**Fig. 5:**
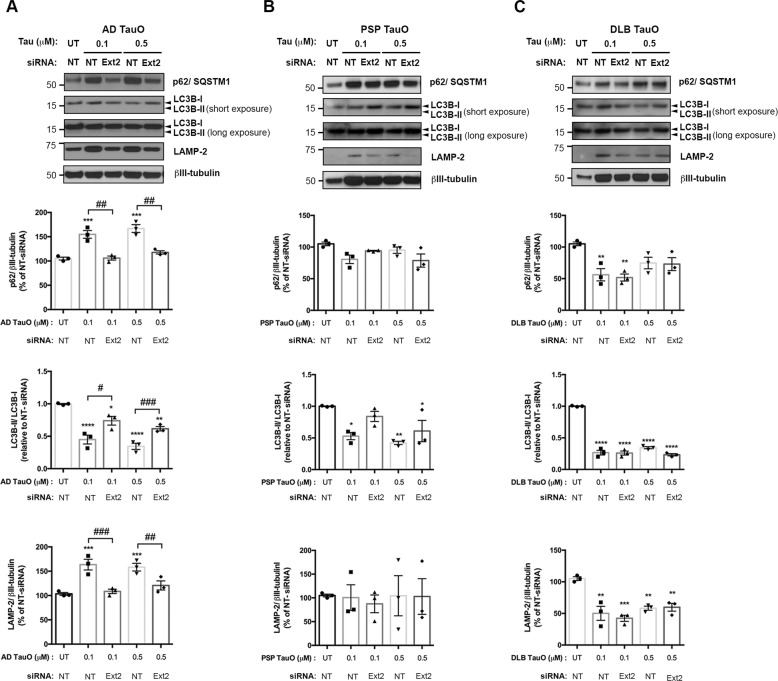
Exogenous tau oligomers mediated alternations of autophagy–lysosomal pathway (ALP) in neurons. Cells were pretreated with NT or Ext2 siRNA followed by the presence or absence of 0.1 μM or 0.5 μM biotin-tagged TauO from AD (**a**), PSP (**b**), or DLB (**c**). All experiments were performed with similar conditions and parameters as for Fig. 4f–h (cells pretreated with NT or Ext2 siRNA followed by the presence or absence of 0.1 μM biotin-tagged TauO), together with samples from cells preincubated with NT or Ext2 siRNA followed by 0.5 μM biotin-tagged TauO. Representative Western blot images revealed the expression of p62 (autophagy receptor), LC3B-II (autophagosome membrane formation), and LAMP-2 (lysosome). Quantification of band intensity shown below was normalized to βIII-tubulin. The same immunoblots probed with loading controls shown in Fig. 4f–h were reused in Fig. 5a–c. **a** AD TauO-exposed cells showed a significant increase in p62 and LAMP-2 levels, yet a drastic decrease was seen in LC3B-II/LC3B-I ratio. Reverse effects were observed in Ext2 siRNA-pretreated group. **b** PSP TauO-treated cells slightly changed the expression of p62, but did not alter LAMP-2 expression. LCB-II/LC3B-I ratio was significantly reduced in PSP TauO-treated, while Ext2 siRNA exposure alleviated the PSP TauO effect without reaching statistical significance. **c** Reductions of p62, LCB-II/LC3B-I ratio, and LAMP-2 were found in DLB TauO-exposed neurons, while the reverse effects from Ext2 siRNA-pretreated were not observed. Statistical analyses were measured by one-way ANOVA with Tukey’s test from three biological independent experiments. Results showed as the value of mean ± SEM, *p < 0.05, **p < 0.01, ***p < 0.001, ****p < 0.0001 vs UT group. ^#^*p* < 0.05, ^##^*p* < 0.01, ^###^*p* < 0.001 vs the TauO-treated group.

Neurodegenerative diseases such as Alzheimer’s, Huntington’s, and Parkinson’s disease are characterized by the deposition of intra- and extracellular proteins aggregates that are not degraded by proteasomes^30^. Therefore, the involvement of autophagy deficiency in neurodegeneration has been observed^31–33^. To determine whether exogenous TauO altered expressions of ALP, which is crucial for misfolded protein degradation^34,35^, cells were treated with two different TauO concentrations (0.1 μM and 0.5 μM) for 18 h. Western blot analysis demonstrated that internalized AD TauO induced increased levels of autophagosome receptors (p62/SQSTM1) (*p* < 0.001) and lysosomes (LAMP-2) (*p* < 0.001). In contrast, an autophagosome membrane-conjugated form (LC3B-II) was disturbed as shown by a decrease ratio of LC3B-II/LC3B-I (membrane-conjugate form/cytosolic form) (*p* < 0.0001), indicating the exogenously applied AD TauO affected autophagosome formation. When TauO internalization was inhibited by Ext2 siRNA, levels of the three proteins were close to UT (*p* < 0.01) (Fig. 5a). In parallel, we did not observe p62 (*p* = 0.0529) and LAMP-2 (*p* = 0.4947) expression changes in response to PSP TauO administration. The LC3B-II/LC3B-I ratio was reduced in PSP TauO-treated group (*p* < 0.01), and it was moderately increased by Ext2 siRNA compared with PSP TauO-treated alone (*p* = 0.0909) (Fig. 5b). In DLB TauO-exposed cells, levels of ALP markers were significantly reduced in respect to UT (*p* < 0.05). Downregulation of Ext2 did not attenuate the DLB TauO effects (*p* = 0.5790) (Fig. 5c). These findings suggest that exogenous AD, PSP, and DLB TauO administration alter the system of protein degradation. This process disturbs the autophagosome formation, and ultimately leads to protein accumulation^36^.

### Downregulation of Ext2 reduced phosphorylated tau levels in neurons treated with exogenous brain-derived tau oligomers

**Fig. 6:**
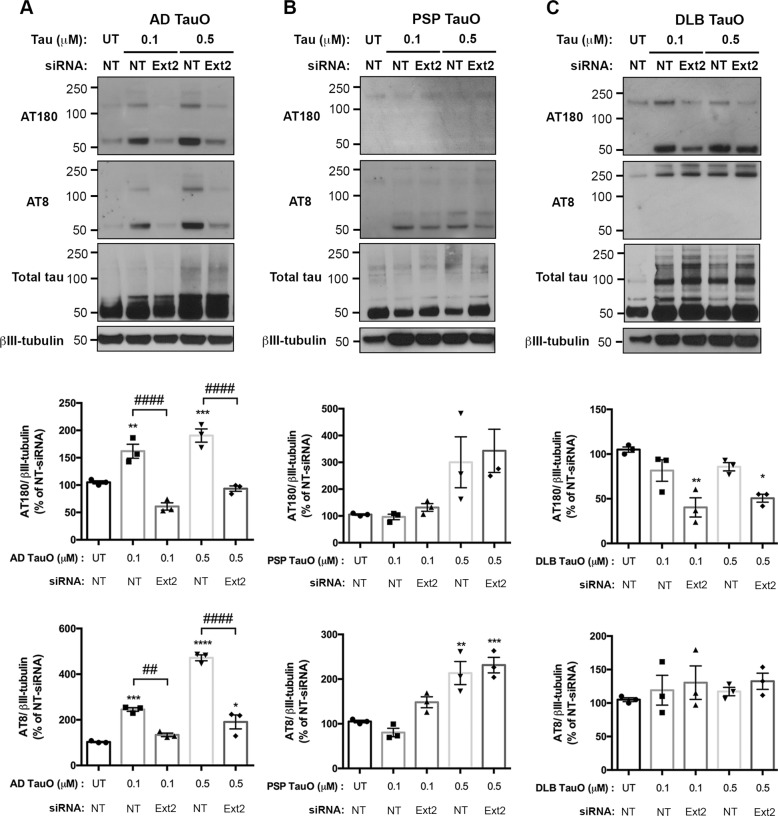
Ext2 knockdown mitigates extracellular tau oligomer-induced phosphorylated tau in neurons. Cells were pretreated with NT or Ext2 siRNA followed by the presence or absence of TauO from AD (**a**), PSP (**b**), or DLB (**c**). All experiments were performed with similar conditions and parameters to experiments indicated in Figs. 4 and 5. Representative Western blot analyses and quantifications showed levels of p-Tau detected by monoclonal antibodies AT180 and AT8, and total tau indicated by total Tau antibody. βIII-tubulin was used for loading control and normalization similar to Figs. 4 and 5. **a** AD TauO-treated cells (0.1 or 0.5 μM) revealed a significant increase of p-Tau (AT180 and AT8) in a concentration-dependent manner, which were reduced in Ext2 siRNA-pretreated group. **b** PSP TauO (0.5 μM) significantly elevated the level of p-Tau (AT8), as well as increased the p-Tau (AT180). **c** DLB TauO-exposed cells altered p-Tau (AT180) levels, while pretreatment of Ext2 siRNA drastically reduced the p-Tau (AT180) expression. Changes were not observed in p-Tau (AT8) after DLB TauO treatment. Statistical analyses were measured by one-way ANOVA with Tukey’s test from three biological independent experiments. Results showed as the value of mean ± SEM, *p < 0.05, **p < 0.01, ***p < 0.001, ****p < 0.0001 vs the UT group. ^##^*p* < 0.01, ^####^*p* < 0.0001 vs the TauO-treated group.

Previous studies have established that extracellular tau aggregates can propagate a misfolded state in a cell externally to internally^37,38^. To address whether exogenous TauO administration would cause phosphorylation-dependent changes of tau in neurons, expressions of various conformations of phosphorylated tau (p-Tau) were investigated. Ext2 siRNA-treated group was compared with a TauO-treated group. Western blot analysis demonstrated that AD TauO at a low concentration (0.1 μM) or higher for 18 h dramatically induced increased levels of p-Tau as determined by monoclonal antibodies (AT180 and AT8) (*p* < 0.001). As expected, Ext2 siRNA pre-exposure reduced the p-Tau levels (*p* < 0.0001) (Fig. 6a). In PSP TauO-applied neurons, levels of p-Tau (Thr231) were mildly changed compared to UT (*p* = 0.4157). In addition, PSP TauO with or without Ext2 siRNA notably enhanced the levels of p-Tau at Ser202 and Thr205 compared with UT (p < 0.01) (Fig. 6b). Conversely, in neurons exposed to DLB TauO studies, Ext2 siRNA significantly reduced the levels of p-Tau (Thr231) in DLB TauO-treated neurons in respect to DLB TauO-treated without Ext2 siRNA and UT (*p* < 0.01). While AT8-positive signals were not changed between the groups (*p* = 0.4186) (Fig. 6c). These results suggest that exogenous TauO from different diseases differentially promote tau phosphorylation in neurons.

## Discussion

Identifying the mechanisms of tau oligomers internalization is essential to understanding how tau pathology initially spreads and leads to pathological progression. Here, we determined the mechanisms of internalization of well-characterized tau oligomers from human tauopathies that we have previously established, including AD^15^, PSP^19^, and DLB TauO^20^. In this study, we found that the primary internalization pathway that plays a critical role in AD and DLB TauO uptake in cortical neurons was HSPG-mediated endocytosis, while other alternative pathways may play a role in PSP TauO uptake. Unsonicated tau fibrils were unable to be internalized to cause toxicity^39^, they, however, were located along the cell membrane. The internalized TauO trafficked along the endosomal–lysosomal system in the early phase of translocation. In the later phase, however, we observed ALP alterations of the autophagosome receptors, indicative of changed autophagosome and lysosome quantity. Blocking the functions of HSPG with Heparin as well as decreasing the expression of HS-synthesizing enzyme, Ext2 by using Ext2 siRNA, reduced the internalization, the changes in ALP, and the levels of phosphorylated tau. This indicates that the primary mechanism for AD and DLB TauO cellular uptake is the HSPG-mediated pathway.

Prion-like transmission of aggregated forms of other amyloid proteins gain entry into cells via macropinocytosis^40–42^. Previous studies showed contradictory findings in different cell models regarding the mechanism for tau internalization. For instance, monomeric tau and tau aggregates entered into human induced-pluripotent stem cells (iPS)-derived neurons via dynamin-mediated and actin polymerization-dependent pathways^43^. In contrast, macropinocytosis is required for uptake of recombinant tau aggregates and short tau fibrils in mouse hippocampal, cortical primary neurons^27^, and human embryonic kidney cells^6^. It is important to note that not only is the internalization mechanism cell-type specific but it is also dependent on distinct protein conformations^7^. In addition, studies suggest that specific binding sites require a precise GAG architecture with defined sulfate moieties in the N- and 6-*O*-positions for tau-HSPG interactions on the cell surface^12,13^. Our findings revealed that AD and DLB TauO were internalized by primary cortical neurons via HSPG-mediated endocytosis, which was prevented by HSPG antagonist as well as HS-synthesizing enzyme Ext2 knockdown.

In neurons, ALP has been mentioned to play a critical role in the degradation processes for cell survival, and has recently received attention in relation to pathogenesis of tauopathies^44^. ALP markers p62, LC3B, and LAMP-2 have frequently demonstrated alterations in expression in AD^36,45,46^. Previously, p62 knockdown mice showed age-dependent NFT accumulations, exhibiting the role of p62 in tauopathies^36^. Our results found similar significance as p62 expression increased in neurons exposed to AD TauO, potentially stimulating pathologic tau degradation. LAMP-2 also shows higher expression when cells were exposed to AD TauO compared with the untreated group. This may be indicative of increased ALP activity, though other studies have reported that several lysosomal storage diseases have greater expression due to lower lysosomal clearance^47^. LC3B-II/LC3B-I ratios have been shown to increase in AD in prior research^45,46^. We observed a decrease in expression when cells were exposed to AD TauO. One explanation of such decrease may be due to the compensation for increased p62 expression considering that p62 will bind to LC3B-II, allowing the cargos to interact with the autophagosome. It is also important to note that the alterations may be due to various factors in our study, such as the duration or the timeframe that neurons were exposed to TauO.

It is well known that pathologic tau is taken up into the neuron; however, the mechanism has yet to be clearly elucidated. Our study implicates tau oligomer uptake via macropinocytosis by inhibiting HSPG function. Mechanistic roles of macropinocytosis in transgenic P301S mice and macropinosome inhibition have also demonstrated^48,49^. As previously discussed, we showed increased expressions of p62, LAMP-2, p-Tau (Thr231 and Ser202, Thr205) and decreased expression of LC3B-II-/LC3B-I when primary neurons were exposed to AD TauO. When cells were knocked down for Ext2 and treated with AD TauO, we noted all expressions reverting to that of the UT group. This may be due to decreased AD TauO uptake via downregulation of macropinocytosis. It is important to note that though LC3B-II/LC3B-I expressions in the Ext2 knockdown group showed similar trend as the untreated group, we still observed a significant alteration in expression. Although our results and other studies indicate a role of macropinocytosis in AD TauO uptake, it is possible that several mechanisms allow for tau to be internalized.

TauO also contributes to the pathology of PSP and DLB^50,51^. In PSP TauO-exposed neurons, a reduction of p62 was noted without significant alterations of LAMP-2 and LC3B-II/LC3B-I expressions. Results support a downregulation of activity in ALP. When primary neurons were exposed to DLB TauO, all ALP markers tested showed a reduction in expression (p62, LAMP-2, LC3B-II/LC3B-I). As increased LAMP-2 expression suggests decreased lysosomal clearance, the decrease in expression may indicate increased clearance and less lysosomal degradation of cargo^47^. From previous studies, p62 loss is a component of hyperphosphorylated tau pathology accumulation and insoluble K63-linked polyubiquitin chain accumulation^51^. Our study also demonstrated increased expression of phosphorylated tau in neurons, implicating that downregulated ALP could be contributing to the accumulation.

Several methods have been used for tau filament preparation in vitro. Heparin has been widely used to induce the recombinant tau into filaments by changing the random coil structure to β-sheet structures^52,53^. To enhance the conformational change, tau filamentation needs to be performed in specific conditions, i.e., speed, rotation, tau/Heparin ratio, the amount of tau monomer, along with a longer period of incubation time^54^. In comparison with our experimental setting, neurons were exposed with Heparin for 30 min prior to treatment with BDTOs at μM range. These factors should not be necessary to enhance BDTOs in cell culture to develop tau fibrils.

In our previous study, we showed that tau interacts with oligomers of other proteins, such as amyloid-β, α-synuclein, and TDP-43, to form hybrid oligomers in AD brains^55^. In postmortem DLB tissues, we found that α-synuclein and tau oligomers form a complex, suggesting that protein interaction at oligomeric levels occurs broadly in the human brain^56^. In PSP, known as a primary tauopathy, argophylic threads and coiled bodies comprised 4R tau have been found in oligodendrocytes^57^. In this study, we found that PSP TauO possessed distinct characteristics of internalization compared with other BDTOs. It is likely due to the molecular heterogeneity of tauopathies, such as isoform composition and deposition of other amyloid aggregates^58^. Similar to our findings, another recent study demonstrated that frontotemporal dementia (FTD)-associated pathological tau oligomers were likely to internalize to neurons by endocytosis^59^. Due to these various factors, it is still unknown if there is a universal internalization mechanism for pathological tau.

Based on flow cytometry analysis, we observed that Heparin blocks most efficiently the internalization of all three BDTOs compared with Dynasore and Nystatin at 1-h incubation of oligomers. Efficiency of Heparin by inhibiting the internalization of AD TauO and DLB TauO was significantly higher than PSP TauO. This indicates that the HSPG-mediated pathway is more selective for AD and DLB TauO compared with PSP TauO. Although, at longer incubation of primary neurons with all three BDTOs, we observed slightly increased internalization of PSP TauO compared with 1-h incubation. However, it requires further investigation to clearly dissect out the selectivity of PSP TauO for HSPG-mediated internalization.

HSPG plays important roles in biological functions in mammalians^60^. In human and mouse AD brains, HSPG is one of the major players that capture cellular Aβ for endocytosis, while the deficiency of HS in neurons disturbed the clearance pathway via lysosomal degradation^61–63^. Similarly, we observed that HSPG is responsible for neuronal internalization of tau oligomers derived from AD and DLB brain tissues. Regarding the therapeutic implications, the disruption of HSPG functions may contribute to modulation of metabolisms not only in adult brains but also causing dysfunction in other systems, such as hereditary multiple exostoses by Ext2 mutation^64^. Given that HSPG interacted with multiple proteins in neurodegenerative diseases, it will be critical to design and identify compounds that specifically inhibit the interaction between tau and HSPG for the treatment of neurodegenerative diseases in the future.

## Supplementary information

Supplementary information

Supplementary Figure Legends

Supplementary Table S1

Supplementary Table S2

Supplementary Table S3

Supplementary Figure S1

Supplementary Figure S2

Supplementary Figure S3

Supplementary Figure S4

Supplementary Figure S5

Supplementary Figure S6

## References

[1] Braak H, Braak E (1991). Demonstration of amyloid deposits and neurofibrillary changes in whole brain sections. Brain Pathol..

[2] Dujardin S (2014). Neuron-to-neuron wild-type tau protein transfer through a trans-synaptic mechanism: relevance to sporadic tauopathies. Acta Neuropathol. Commun..

[3] Liu L (2012). Trans-synaptic spread of tau pathology in vivo. PLoS ONE.

[4] Kaufman SK (2016). Tau prion strains dictate patterns of cell pathology, progression rate, and regional vulnerability in vivo. Neuron.

[5] Xu D, Esko JD (2014). Demystifying heparan sulfate-protein interactions. Annu. Rev. Biochem.

[6] Holmes BB (2013). Heparan sulfate proteoglycans mediate internalization and propagation of specific proteopathic seeds. Proc. Natl Acad. Sci. USA.

[7] Ihse E (2017). Cellular internalization of alpha-synuclein aggregates by cell surface heparan sulfate depends on aggregate conformation and cell type. Sci. Rep..

[8] Kanekiyo T (2011). Heparan sulphate proteoglycan and the low-density lipoprotein receptor-related protein 1 constitute major pathways for neuronal amyloid-beta uptake. J. Neurosci..

[9] Zhao J (2017). Glycan determinants of heparin-tau interaction. Biophys. J..

[10] Inatani M, Yamaguchi Y (2003). Gene expression of EXT1 and EXT2 during mouse brain development. Brain Res Dev. Brain Res..

[11] Takeuchi K (2013). Chondroitin sulphate N-acetylgalactosaminyl-transferase-1 inhibits recovery from neural injury. Nat. Commun..

[12] Rauch JN (2018). Tau internalization is regulated by 6-O sulfation on heparan sulfate proteoglycans (HSPGs). Sci. Rep..

[13] Stopschinski BE (2018). Specific glycosaminoglycan chain length and sulfation patterns are required for cell uptake of tau versus alpha-synuclein and beta-amyloid aggregates. J. Biol. Chem..

[14] Lasagna-Reeves CA, Glabe CG, Kayed R (2011). Amyloid-beta annular protofibrils evade fibrillar fate in Alzheimer disease brain. J. Biol. Chem..

[15] Lasagna-Reeves CA (2012). Alzheimer brain-derived tau oligomers propagate pathology from endogenous tau. Sci. Rep..

[16] Patterson KR (2011). Characterization of prefibrillar tau oligomers in vitro and in Alzheimer disease. J. Biol. Chem..

[17] Sengupta U, Carretero-Murillo M, Kayed R (2018). Preparation and characterization of Tau oligomer strains. Methods Mol. Biol..

[18] Lasagna-Reeves CA, Castillo-Carranza DL, Guerrero-Muoz MJ, Jackson GR, Kayed R (2010). Preparation and characterization of neurotoxic tau oligomers. Biochemistry.

[19] Gerson JE (2014). Characterization of tau oligomeric seeds in progressive supranuclear palsy. Acta Neuropathol. Commun..

[20] Lasagna-Reeves CA (2014). The formation of tau pore-like structures is prevalent and cell specific: possible implications for the disease phenotypes. Acta Neuropathol. Commun..

[21] Lo Cascio F (2019). Toxic tau oligomers modulated by novel curcumin derivatives. Sci. Rep..

[22] Perea JR (2019). Extracellular monomeric tau is internalized by astrocytes. Front. Neurosci..

[23] Usenovic M (2015). Internalized tau oligomers cause neurodegeneration by inducing accumulation of pathogenic tau in human neurons derived from induced pluripotent stem cells. J. Neurosci..

[24] Wauters, M., Wattiez, R. & Ris, L. Internalization of the extracellular full-length tau inside Neuro2A and cortical cells is enhanced by phosphorylation. *Biomolecules* **6**, 36 (2016). 10.3390/biom6030036 PMC5039422 27548242

[25] Xie J (2006). Novel fiber-dependent entry mechanism for adenovirus serotype 5 in lacrimal acini. J. Virol..

[26] Lo Cascio F, Kayed R (2018). Azure C targets and modulates toxic tau oligomers. ACS Chem. Neurosci..

[27] Wu JW (2013). Small misfolded tau species are internalized via bulk endocytosis and anterogradely and retrogradely transported in neurons. J. Biol. Chem..

[28] Zhou L (2017). Tau association with synaptic vesicles causes presynaptic dysfunction. Nat. Commun..

[29] Hansen TE, Johansen T (2011). Following autophagy step by step. BMC Biol..

[30] Levine B, Kroemer G (2008). Autophagy in the pathogenesis of disease. Cell.

[31] Adams J (2019). Autophagy-lysosome pathway alterations and alpha-synuclein up-regulation in the subtype of neuronal ceroid lipofuscinosis, CLN5 disease. Sci. Rep..

[32] Elia LP, Mason AR, Alijagic A, Finkbeiner S (2019). Genetic regulation of neuronal progranulin reveals a critical role for the autophagy-lysosome pathway. J. Neurosci..

[33] Metz KA (2018). KCTD7 deficiency defines a distinct neurodegenerative disorder with a conserved autophagy-lysosome defect. Ann. Neurol..

[34] Jackson MP, Hewitt EW (2016). Cellular proteostasis: degradation of misfolded proteins by lysosomes. Essays Biochem.

[35] Thellung, S., Corsaro, A., Nizzari, M., Barbieri, F. & Florio, T. Autophagy activator drugs: a new opportunity in neuroprotection from misfolded protein toxicity. *Int. J. Mol. Sci.* **20**, 901 (2019). 10.3390/ijms20040901 PMC6412775 30791416

[36] Caccamo A, Ferreira E, Branca C, Oddo S (2017). p62 improves AD-like pathology by increasing autophagy. Mol. Psychiatry.

[37] Frost B, Jacks RL, Diamond MI (2009). Propagation of tau misfolding from the outside to the inside of a cell. J. Biol. Chem..

[38] Sanders DW (2014). Distinct tau prion strains propagate in cells and mice and define different tauopathies. Neuron.

[39] Ghag G (2018). Soluble tau aggregates, not large fibrils, are the toxic species that display seeding and cross-seeding behavior. Protein Sci..

[40] Munch C, O’Brien J, Bertolotti A (2011). Prion-like propagation of mutant superoxide dismutase-1 misfolding in neuronal cells. Proc. Natl Acad. Sci. USA.

[41] Wesen E, Jeffries GDM, Matson Dzebo M, Esbjorner EK (2017). Endocytic uptake of monomeric amyloid-beta peptides is clathrin- and dynamin-independent and results in selective accumulation of Abeta(1-42) compared to Abeta(1-40). Sci. Rep..

[42] Zeineddine R, Yerbury JJ (2015). The role of macropinocytosis in the propagation of protein aggregation associated with neurodegenerative diseases. Front. Physiol..

[43] Evans LD (2018). Extracellular monomeric and aggregated tau efficiently enter human neurons through overlapping but distinct pathways. Cell Rep..

[44] Zare-Shahabadi A, Masliah E, Johnson GV, Rezaei N (2015). Autophagy in Alzheimer’s disease. Rev. Neurosci..

[45] Armstrong A (2014). Lysosomal network proteins as potential novel CSF biomarkers for Alzheimer’s disease. Neuromolecular Med..

[46] Coffey EE, Beckel JM, Laties AM, Mitchell CH (2014). Lysosomal alkalization and dysfunction in human fibroblasts with the Alzheimer’s disease-linked presenilin 1 A246E mutation can be reversed with cAMP. Neuroscience.

[47] Hua CT, Hopwood JJ, Carlsson SR, Harris RJ, Meikle PJ (1998). Evaluation of the lysosome-associated membrane protein LAMP-2 as a marker for lysosomal storage disorders. Clin. Chem..

[48] Falcon B (2015). Conformation determines the seeding potencies of native and recombinant tau aggregates. J. Biol. Chem..

[49] Lewis J, Dickson DW (2016). Propagation of tau pathology: hypotheses, discoveries, and yet unresolved questions from experimental and human brain studies. Acta Neuropathol..

[50] Koga S (2017). Cognitive impairment in progressive supranuclear palsy is associated with tau burden. Mov. Disord..

[51] Tanji K (2015). p62 deficiency enhances alpha-Synuclein pathology in mice. Brain Pathol..

[52] Berriman J (2003). Tau filaments from human brain and from in vitro assembly of recombinant protein show cross-beta structure. Proc. Natl Acad. Sci. USA.

[53] von Bergen M (2001). Mutations of tau protein in frontotemporal dementia promote aggregation of paired helical filaments by enhancing local beta-structure. J. Biol. Chem..

[54] Zhang, W. et al. Heparin-induced tau filaments are polymorphic and differ from those in Alzheimer’s and Pick’s diseases. *eLife* **8**, e43584 (2019). 10.7554/eLife.43584 PMC6375701 30720432

[55] Guerrero-Munoz MJ, Castillo-Carranza DL, Kayed R (2014). Therapeutic approaches against common structural features of toxic oligomers shared by multiple amyloidogenic proteins. Biochem. Pharm..

[56] Sengupta U (2015). Pathological interface between oligomeric alpha-synuclein and tau in synucleinopathies. Biol. Psychiatry.

[57] Arima K (2006). Ultrastructural characteristics of tau filaments in tauopathies: immuno-electron microscopic demonstration of tau filaments in tauopathies. Neuropathology.

[58] Dujardin S (2018). Different tau species lead to heterogeneous tau pathology propagation and misfolding. Acta Neuropathol. Commun..

[59] Karikari TK (2019). Distinct conformations, aggregation and cellular internalization of different tau strains. Front. Cell Neurosci..

[60] Bishop JR, Schuksz M, Esko JD (2007). Heparan sulphate proteoglycans fine-tune mammalian physiology. Nature.

[61] Kanekiyo T (2013). Neuronal clearance of amyloid-beta by endocytic receptor LRP1. J. Neurosci..

[62] Kanekiyo T, Xu H, Bu G (2014). ApoE and Abeta in Alzheimer’s disease: accidental encounters or partners?. Neuron.

[63] Liu CC (2016). Neuronal heparan sulfates promote amyloid pathology by modulating brain amyloid-beta clearance and aggregation in Alzheimer’s disease. Sci. Transl. Med.

[64] Narvid J (2009). Of brain and bone: the unusual case of Dr. A. Neurocase.

