# Supplementary material for: Internalization mechanisms of brain-derived tau oligomers from patients with Alzheimer’s disease, progressive supranuclear palsy and dementia with Lewy bodies
Source: Cell Death Dis. 2020 May 4;11(5):314. doi: 10.1038/s41419-020-2503-3 (PMC7198578; doi:10.1038/s41419-020-2503-3)
Supplement: Supplementary file 1 — Supplementary information [file 41419_2020_2503_MOESM1_ESM.docx]

**Supplementary Information**

**Supplementary Materials and Methods**

**Characterization of brain-derived tau oligomers**

The characterization was performed by various methods as previously described^1^. The morphology of immunoprecipitated structures was assessed as previously described by Atomic Force Microscopy (AFM) using a non-contact tapping method (ScanAsyst-air) with a Multimode 8 AFM machine (Veeco, CA). The size was determined by Western blot using Tau-5 antibody. Size-exclusion chromatography analysis and purifications were performed using a LC-6AD Shimadsu high-performance liquid chromatography (HPLC) system fitted with a TSK-GEL G3000 SWXL (30 cm x 7.8 mm) column, Supelco-808541. PBS, pH 7.4, was used as the mobile phase, flow rate 0.5 ml/min. Gel filtration standard (Bio-Rad; 51-1901) was used for calibrations. Thioflavin T (ThT) assay**:** samples were prepared using 1 μl of protein sample and 250 μl of 5 μM ThT, 50 mM glycine-NaOH buffer (pH 8.5) in clear bottom 96-well black plates. The ThT fluorescence intensity of each sample was recorded using a POLARstar OMEGA plate reader (BMG Labtechnologies, Melbourne, VIC, Australia) with Fluorescence was measured at λ-emission 490 nm upon excitation at λ-excitation 440 nm. For the Bis-ANS assay, samples were prepared using 1 μl of protein sample and 250 μl of 10 μM Bis-ANS, 100 mM glycine-NaOH buffer (pH 7.4) in clear bottom 96-well black plates. The ThT fluorescence intensity of each sample was recorded using a POLARstar OMEGA plate reader (BMG Labtechnologies, Melbourne, VIC, Australia) with λ-emission 520 nm upon excitation at λ-excitation 380 nm. Western blot analysis was performed as we recently described^2^, PBS-soluble fractions of brain extracts were run on Bis-Tris SDS-PAGE gels and subsequently transferred onto nitrocellulose. After blocking overnight at 4°C with 10% nonfat dried milk, membranes were probed for 1 h at room temperature with anti-tau oligomer antibody T22^3^.

**Preparation of recombinant tau species**

Recombinant tau protein [tau-441 (2N4R) MW 45.9 kDa] was expressed and purified as described previously^4^. It was treated with 8 M urea to obtain monomeric tau and dialyzed overnight against molecular grade water (pH 7.4). Tau was aliquoted and lyophilized. For preparation of oligomers, lyophilized tau was resuspended in 1ml of 1X PBS and diluted to make a final concentration of 0.3 mg/ml. Aliquots of a monomeric tau solution were prepared in sterile water with 1×PBS and incubated with Heparin (15 kDa) in a 1:4 ratio of Heparin to TauM^3^ at 37°C for 3–5 days. The tau-Heparin mixture was rotated constantly using a rotary shaker at a speed of 30 rpm. The characterization of tau oligomers and fibrils was performed as described previously^1^.

**Seeding assay**

Recombinant tau 4R monomers were obtained by dissolving lyophilized pellets of recombinant 4R tau at 1 mg/ml concentration in PBS^5^ and seeded with AD, PSP, or DLB-brain derived tau oligomers. The oligomer-monomer mixture was made at a ratio of 1:100 (w/w) with gentle agitation at room temperature for 2 days. After seeding, aliquots were taken and immediately used for Western blotting using T22 and Tau-13 antibody as well as AFM analysis. Total protein concentration was determined using the bicinchoninic acid protein assay (Pierce) and stored at -20°C until use.

**Atomic force microscopy**

The amount of 10 µl of BDTOs, recombinant human tau, or fibrils were applied onto a freshly-cleaved mica disk and allowed to absorb. Mica was then washed with 100 µl of deionized water and air-dried. Samples were analyzed by a non-contact tapping method using a Multimode 8 AFM machine (Veeco, Plainview, NY).

**Proteinase K digestion**

Procedures for proteinase K digestion were previously described^6^.

**Tau RD P301S biosensor cell culture and treatment**

Tau RD P301S biosensor cells (ATCC, CRL-3275) were culture in DMEM supplemented with 10% FBS, 100 μg/ml penicillin, and 100 μg/ml streptomycin. Cultures were maintained in a humidified atmosphere of 5% CO_2_ at 37°C. For prion-like activity assay, the cells were plated on poly-L-Lysine-coated coverslips at a density of 1 x 10^5^ cells/well in a 24-well plate. After 18 h, cells (generation 1) were transduced with BDTOs/Lipofectamine 2000 (Invitrogen, 11668-027) mixture in Opti-Mem (Gibco, 31985-070) [100 nM BDTOs + 6 μl Lipofectamine 2000 + Opti-Mem] for a total volume of 50 μl/well. BTDO/liposome mixture was incubated at RT for 30 min before adding it to cells. Cells were incubated with transduction complexes for 24 h. For BDTO propagation through the next cell generation, transduced cells (generation 1) were lysed with 0.05% Triton X-100 (Sigma, T8787) mixed with 2% protease inhibitor (Sigma, P8340). Total cell lysates were collected from centrifugation at 13,000 rpm, 4°C for 10 min, followed by protein quantification using Pierce^TM^ BCA protein assay kit. Cells (generation 2) were transduced with total lysate/Lipofectamine 2000 mixture in Opti-Mem [1.5 μg total lysates + 6 μl Lipofectamine 2000 + Opti-Mem]. The mixture was incubated at RT for 30 min before adding to cells for 24 h. For the effect of HSPG on the seeding activity, BDTOs (100 nM) were mixed with Heparin (1 μg/ml) in Opti-Mem at 4°C, overnight. Cells were treated with BDTOs or BDTOs/Heparin mixture for 24 h. After treatment, cells were multiple washed with PBS, fixed with 4% formaldehyde for 15 min at RT, and mounted with Prolong Gold mounting media.

**Cytotoxicity**

Neurons at a density of 3 x 10^4^ cells/well in a 96-well plate were pre-treated with 200 μg/ml Heparin for 30 min. AD, PSP, or DLB TauO (0.5 μM monomer equivalent or 24 μg/ml) were added in 100 μl neurobasal media without phenol red. After incubation for 24 h at 37°C, lactate dehydrogenase (LDH) release of culture media was measured using Cytotoxicity Detection kit ^PLUS^ (Roche; 04744926001) according to the manufacturer's directions. Each data point was determined in triplicate of three independent experiments.

**Supplementary Results**

**Distinct biophysical and biochemical characteristics of BDTOS**

BDTOs were purified from postmortem brain tissues of patients with AD, PSP and DLB, and the conformations of tau samples were verified by atomic force microscopy (Fig. S1A), and the size distribution measurements using HPLC (Fig. S1B). Proteinase K enzyme digestion of tau (Fig. S1C) displayed a different fragmentation pattern using Western blotting to detect Tau-5 immunoreactivity, indicating the different tau conformations.

**Prion-like activity of the brain-derived tau oligomers**

Next, we determined the prion-like activity of the BDTOs which is capable of transmission into living cells, propagation through multiple generations, extraction and re-introduction to naïve cells ^7^. Tau RD P301S biosensor cells were used to detect the seeding activity of BDTOs after 24 h. We found that BDTOs from AD, PSP, and DLB illustrated the seeding activity in cells in generation 1 (Fig. S2A, left panel). In addition, cell lysates from first generation were able to continue the seeding activity through cells in generation 2 (Fig. S2A, right panel), suggesting the prion-like activity of the BDTOs in our study.

**Inhibitory effect of Heparin-BDTOs complex on the neuronal internalization**

To investigate the BDTOs internalization using immunocytochemistry, we performed Heparin pre-incubation with BDTOs at 4°C overnight ^8^ followed by neuronal treatment for 1 and 5 h. Results showed that Heparin-BDTOs complex treatment not only inhibited the internalization similar to results from Heparin pre-treated neurons prior to BDTOs exposure, but also enhanced larger aggregation of PSP and DLB TauO binding outside the cells (Fig. S4).

**Heparin inhibited neuronal internalization of recombinant tau oligomer**

In addition to the study of TauO from brain-derived, we also performed experiments with recombinant tau. Heparin pre-treated neurons followed by exposure with 3R tau (Fig. S5A), 4R tau (Fig. S5B), or 3R/4R TauO (1:1) mixture (Fig. S5C) was determined using immunocytochemistry. Results showed that Heparin inhibited the neuronal uptake of the two tau isoforms treated individually as well as the mixture of the 3R and 4R TauO after 1 h. As the results of Heparin treatment, we found the recombinant tau formed high aggregates and bound outside the cells.

**Roles of HSPG on prion-like activity of BDTOs**

We further investigated the role of HSPG on the prion-like activity of BDTOs. Tau RD P301S biosensor cells were used to detect the seeding activity in the present or absent of Heparin after 24 h treatment. Regarding the role of HSPG on BDTO internalization, we did not use lipofectamine 2000 as a vehicle. Results showed that BDTOs/Heparin mixture showed drastic reduction of seeding activity (Fig. S6A) compared to BDTOs-treated group. Quantification of FRET-positive cells represented the significant reduction of seeding activity observed on the three different BDTOs in the presence of Heparin (Fig. S6B-D).

**References**

1 Lasagna-Reeves, C. A., Castillo-Carranza, D. L., Guerrero-Muoz, M. J., Jackson, G. R. & Kayed, R. Preparation and characterization of neurotoxic tau oligomers. *Biochemistry* **49**, 10039-10041, (2010).

2 Kanekiyo, T., Xu, H. & Bu, G. ApoE and Abeta in Alzheimer's disease: accidental encounters or partners? *Neuron* **81**, 740-754, (2014).

3 Lasagna-Reeves, C. A. et al*.* Identification of oligomers at early stages of tau aggregation in Alzheimer's disease. *FASEB J* **26**, 1946-1959, (2012).

4 Margittai, M. & Langen, R. Template-assisted filament growth by parallel stacking of tau. *Proc Natl Acad Sci U S A* **101**, 10278-10283, (2004).

5 Lasagna-Reeves, C. A., Glabe, C. G. & Kayed, R. Amyloid-beta annular protofibrils evade fibrillar fate in Alzheimer disease brain. *J Biol Chem* **286**, 22122-22130, (2011).

6 Sengupta, U., Carretero-Murillo, M. & Kayed, R. Preparation and characterization of tau oligomer strains. *Methods Mol Biol* **1779**, 113-146, (2018).

7 Sanders, D. W. et al*.* Distinct tau prion strains propagate in cells and mice and define different tauopathies. *Neuron* **82**, 1271-1288, (2014).

8 Stopschinski, B. E. et al*.* Specific glycosaminoglycan chain length and sulfation patterns are required for cell uptake of tau versus alpha-synuclein and beta-amyloid aggregates. *J Biol Chem* **293**, 10826-10840, (2018).
