# Supplementary material for: Internalization mechanisms of brain-derived tau oligomers from patients with Alzheimer’s disease, progressive supranuclear palsy and dementia with Lewy bodies
Source: Cell Death Dis. 2020 May 4;11(5):314. doi: 10.1038/s41419-020-2503-3 (PMC7198578; doi:10.1038/s41419-020-2503-3)
Supplement: Supplementary file 2 — Supplementary Figure Legends [file 41419_2020_2503_MOESM2_ESM.docx]

**Supplementary Figure Legends**

**Fig. S1 Distinct biophysical and biochemical characteristics of BDTOs from AD, PSP and DLB human brains.**

(**A**) Representative atomic force microscopy images showed tau oligomers from AD, PSP, DLB human brain-derived, recombinant human tau 4R, and fibrils. Scale bar; 200 nm.

(**B**) HPLC chromatogram of brain-derived oligomers illustrated major peaks corresponding to higher aggregates approximately ranging from 270-670 kDa. Brain-derived tau from AD and DLB showed peaks corresponding to dimers and trimers (~100 - 250 kDa). AD and PSP brain-derived tau also showed minor monomer peaks (~40-75), but DLB did not show any monomer peaks.

(**C**) Proteinase K digestion (1 μg/ml) followed by Western blot analysis with Tau-5 antibody for total tau showed different fragmentation patterns between tau strains.

**Fig. S2 Prion-like activity of brain-derived tau oligomers.**

(**A**) Tau RD P301S biosensor cells were used to determine the seeding activity of BDTOs. Left panel showed cells (generation 1) treated with lipofectamine 2000 (vehicle; veh) or 100 nM BDTO/liposome mixture for 24 h. Right panel showed cells (generation 2) treated with 1.5 μg cell lysates (generation 1)/liposome mixture for 24 h. FRET-positive cells showed bright green signals indicating tau aggregate formation. Representative images with high magnification showed on the left corners of the main figures. Scale bar; 20 μm.

(**B, C**) Quantification of FRET-positive cells in generation 1 (**B**) and generation 2 (**C**). Images were taken from 40x magnification of three random fields in duplicate. Percentage of FRET-positive cells were calculated from total FRET-positive cells divided by total DAPI-positive cells (data not show). Scatter plot represented values of mean ± SEM.

**Fig. S3 Tau fibrils were not internalized into cortical neurons.**

(**A**) Cortical neurons were exposed to tau fibrils for 1 h without Heparin pretreatment. Tau fibrils were found along the cell membrane but were not internalized into cells. Fixed cells were immunolabeled with wheat germ agglutinin (WGA) for cell membrane (red) and Tau-13 for total tau (green), and DAPI (blue) for nuclei. Scale bar; 10 μm.

(**B**) LDH release was measured in cells treated with 0.5 μM TauO from AD, PSP, or DLB for 24 h. Pre-treatment with 200 μg/ml Heparin inhibited LDH ­release from all BDTOs treatment. Bar graphs represent levels of LDH (O.D. 490 nm), shown as the value of mean ± SEM of BDTOs from three different brains. Statistical analyses were calculated by one-way ANOVA with Tukey’s multiple comparison test. **p < 0.01, ****p < 0.0001 vs vehicle control. ^####^ p < 0.0001 vs BDTO-treated without Heparin pretreatment.

**Fig. S4 Inhibitory effect of Heparin-BDTOs complex on the neuronal internalization.**

(**A**) Primary neurons were treated with or without Heparin pre-incubated AF568-tagged TauO from AD, PSP, or DLB for 1 h or 5 h. Cells were fixed and immunostained for a mature neuronal marker (βIII-tubulin, blue) and an early endosomal marker (Rab5, green). Representative images indicate AF568-tagged TauO co-localized to early endosomes, indicated by arrows. Insets show high magniﬁcation of the area indicated in the main image. Scale bar is indicated.

**Fig. S5 Heparin inhibited neuronal internalization of recombinant tau oligomers.**

(**A-C**) Neurons were exposed to AF488-3R TauO (green) (**A**), AF568-4R TauO (**B**), or AF488-3RTauO mixed with AF568-4R TauO (**C**) for 1 h with or without Heparin pre-treatment. Recombinant TauO was found to form higher aggregates, but was not internalized into cells. Fixed cells were immunolabeled with a mature neuronal marker (βIII-tubulin, blue). Scale bar; 10 μm.

(**D-F**) Analysis of integrated fluorescent intensity of internalized AF488-3R TauO (**D**), AF568-4R TauO (**E**), or AF488-3RTauO mixed with AF568-4R TauO (**F**). Each treatment group was randomly imaged in five different regions of interest and performed in triplicate.

Image analyses were calculated by One-way ANOVA with Tukey’s multiple comparison test. Results showed as the value of mean ± SEM, **p < 0.01, ***p < 0.001 vs Heparin untreated.

**Fig. S6 Roles of HSPG on prion-like activity of BDTOs.**

(**A**) Heparin (1 μg/ml) drastically reduced the seeding activity of BDTOs (100 nM) from AD, PSP, and DLB in Tau biosensor cells after 24 h. Scale bar; 20 μm.

(**B-D**) Quantification of FRET-positive cells from treatment of BDTO-treated with (+) or without (-) Heparin. Percentage of FRET-positive cells was calculated from total FRET-positive cells divided by DAPI-positive cells (data not show). Scatter plot represented values of mean ± SEM from 40x magnification of three random fields in duplicate. Statistical analyses were calculated by one-way ANOVA with Tukey’s multiple comparison test. **p < 0.01, ***p < 0.001, ****p < 0.0001 vs UT. ^#^p < 0.05, ^##^p < 0.01, ^####^p < 0.0001 vs BDTO-treated (-Heparin).
