# Supplementary material for: Internalization mechanisms of brain-derived tau oligomers from patients with Alzheimer’s disease, progressive supranuclear palsy and dementia with Lewy bodies
Source: Cell Death Dis. 2020 May 4;11(5):314. doi: 10.1038/s41419-020-2503-3 (PMC7198578; doi:10.1038/s41419-020-2503-3)
Supplement: Supplementary file 3 — Supplementary Table S1 [file 41419_2020_2503_MOESM3_ESM.docx]

**Supplementary Table S1**

**Summary of human cases examined in this study**

| **Pathology/ Brain #** | **Age** | **Gender** | **Post-mortem interval (h)** | **Brain area** | **Braak stage** |
| --- | --- | --- | --- | --- | --- |
| AD1 | 77 | Male | 4.5 | Frontal cortex | VI |
| AD2 | 83 | Male | 3.5 | Frontal cortex | VI |
| AD3 | 82 | Female | 3.1 | Frontal cortex | VI |
| PSP1 | 72 | Male | 11 | Frontal cortex | NA |
| PSP2 | 73 | Male | 8.5 | Frontal cortex | NA |
| PSP3 | 79 | Male | 12 | Frontal cortex | NA |
| DLB1 | 76 | Female | 19 | Frontal cortex | NA |
| DLB2 | 67 | Female | 12 | Frontal cortex | NA |
| DLB3 | 72 | Male | 24 | Frontal cortex | NA |
