# Supplementary material for: Internalization mechanisms of brain-derived tau oligomers from patients with Alzheimer’s disease, progressive supranuclear palsy and dementia with Lewy bodies
Source: Cell Death Dis. 2020 May 4;11(5):314. doi: 10.1038/s41419-020-2503-3 (PMC7198578; doi:10.1038/s41419-020-2503-3)
Supplement: Supplementary file 4 — Supplementary Table S2 [file 41419_2020_2503_MOESM4_ESM.docx]

**Supplementary Table S2**

**All target sequences in siRNA transfection**

| **siRNA targeting gene** | **Targeting sequence** | **Resource** |
| --- | --- | --- |
| Non target | 1. UGGUUUACAUGUCGACUAA | D-001910-01-05 |
| Ext2 | 1: GUAGAAUGCAUACGUGUUU  2: CUGUCCUCUACAAAGAUGA  3. CUUCUAUCACAAGUAUUUU  4. GUUUCUUCCCUUACGACGA | E-065097-00-0005 |
