# Supplementary material for: Internalization mechanisms of brain-derived tau oligomers from patients with Alzheimer’s disease, progressive supranuclear palsy and dementia with Lewy bodies
Source: Cell Death Dis. 2020 May 4;11(5):314. doi: 10.1038/s41419-020-2503-3 (PMC7198578; doi:10.1038/s41419-020-2503-3)
Supplement: Supplementary file 5 — Supplementary Table S3 [file 41419_2020_2503_MOESM5_ESM.docx]

**Supplementary Table S3**

**List of primers for RT-qPCR**

| **Gene** | **Accession number** | **Primer sequences** |
| --- | --- | --- |
| *Ext2* | NM_001355075 | Sense primer: 5’-TGCTGGTGGTCTGGAATAATCAG-3’  Anti-sense primer: 5’-ACTTAGCTTGTTTTCTGCAGTCCTC-3’ |
