# Supplementary figures and images for: Internalization mechanisms of brain-derived tau oligomers from patients with Alzheimer’s disease, progressive supranuclear palsy and dementia with Lewy bodies
Source: Cell Death Dis. 2020 May 4;11(5):314. doi: 10.1038/s41419-020-2503-3 (PMC7198578; doi:10.1038/s41419-020-2503-3)

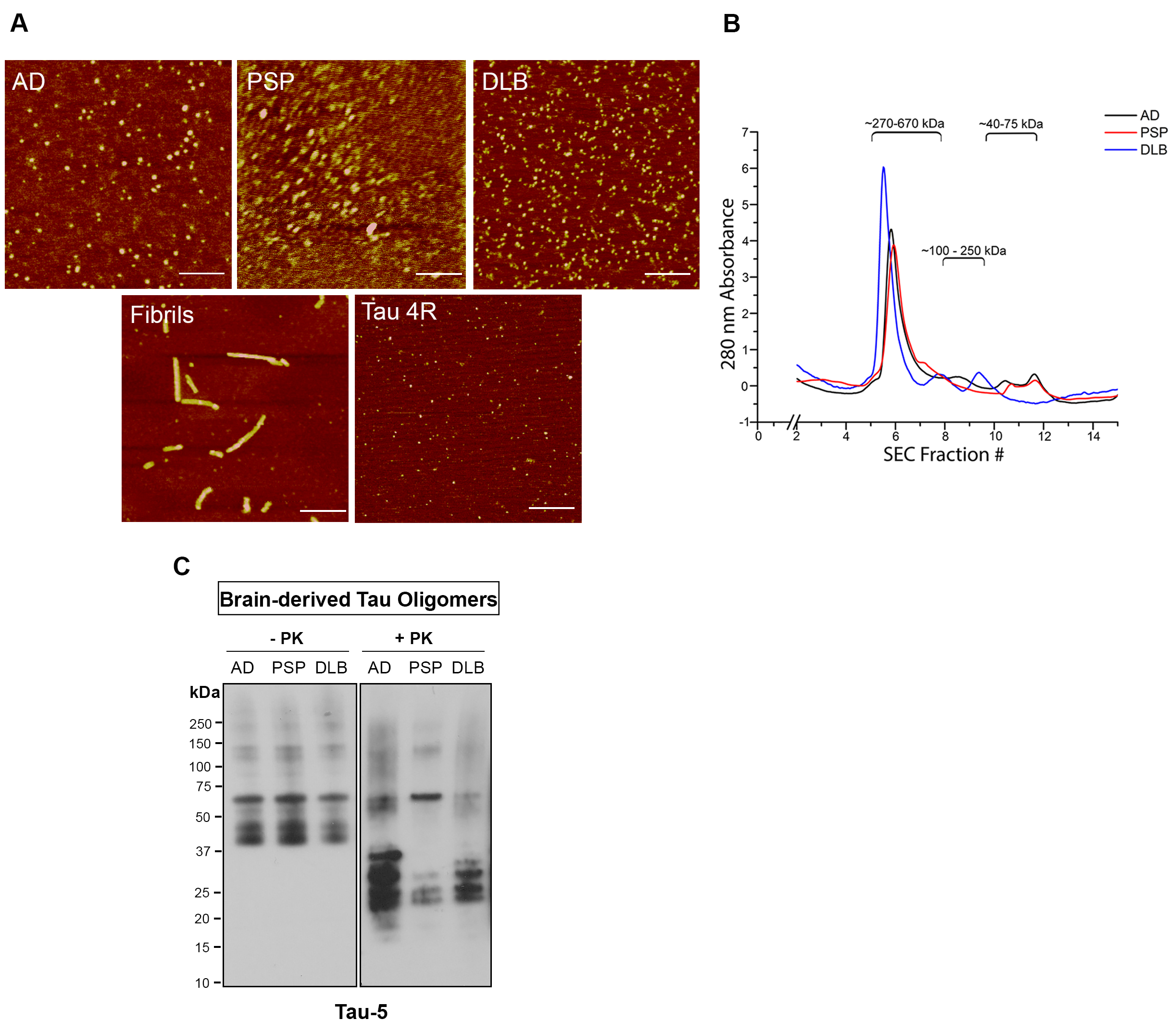

Supplement: Supplementary file 6 — Supplementary Figure S1 [file 41419_2020_2503_MOESM6_ESM.tif]

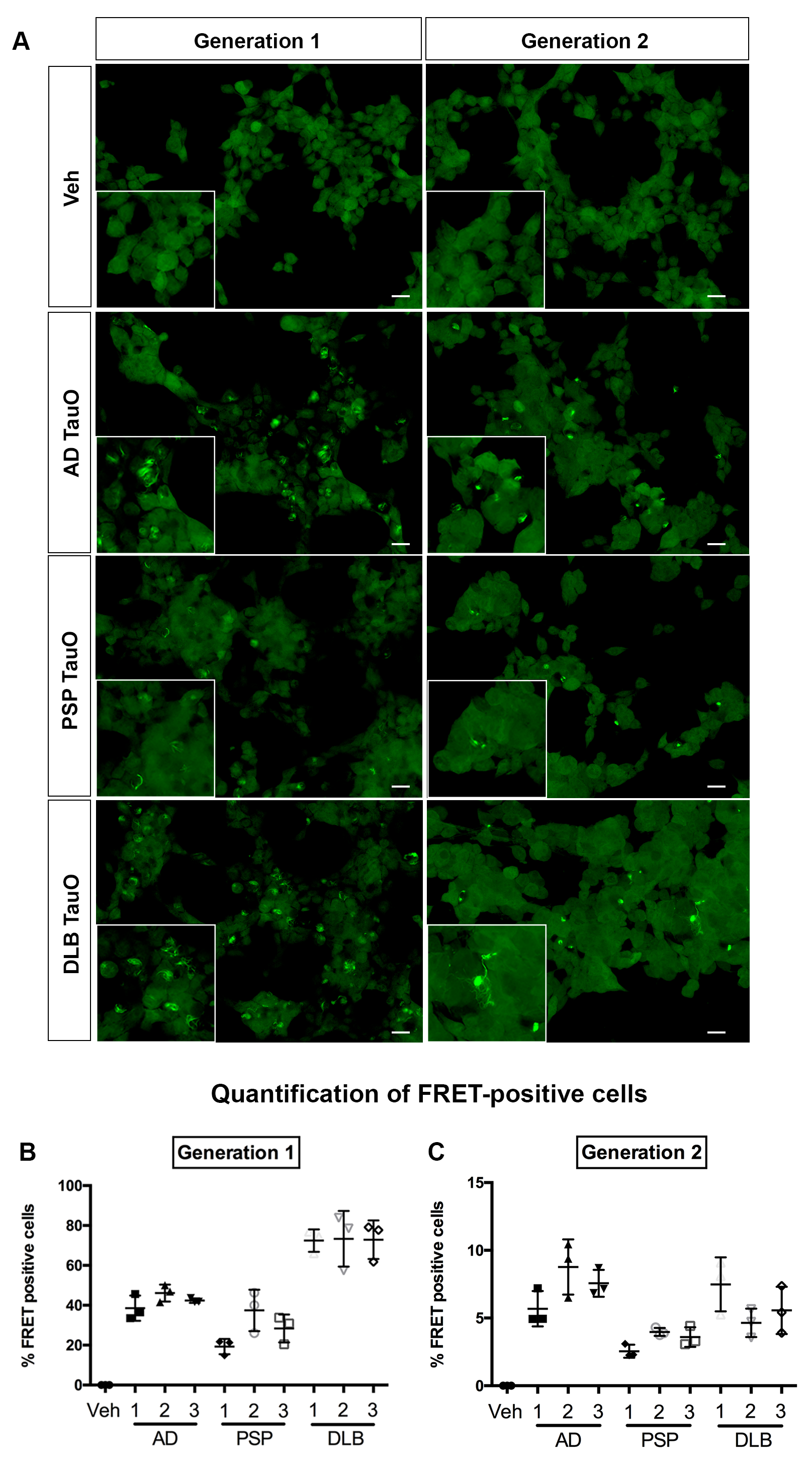

Supplement: Supplementary file 7 — Supplementary Figure S2 [file 41419_2020_2503_MOESM7_ESM.tif]

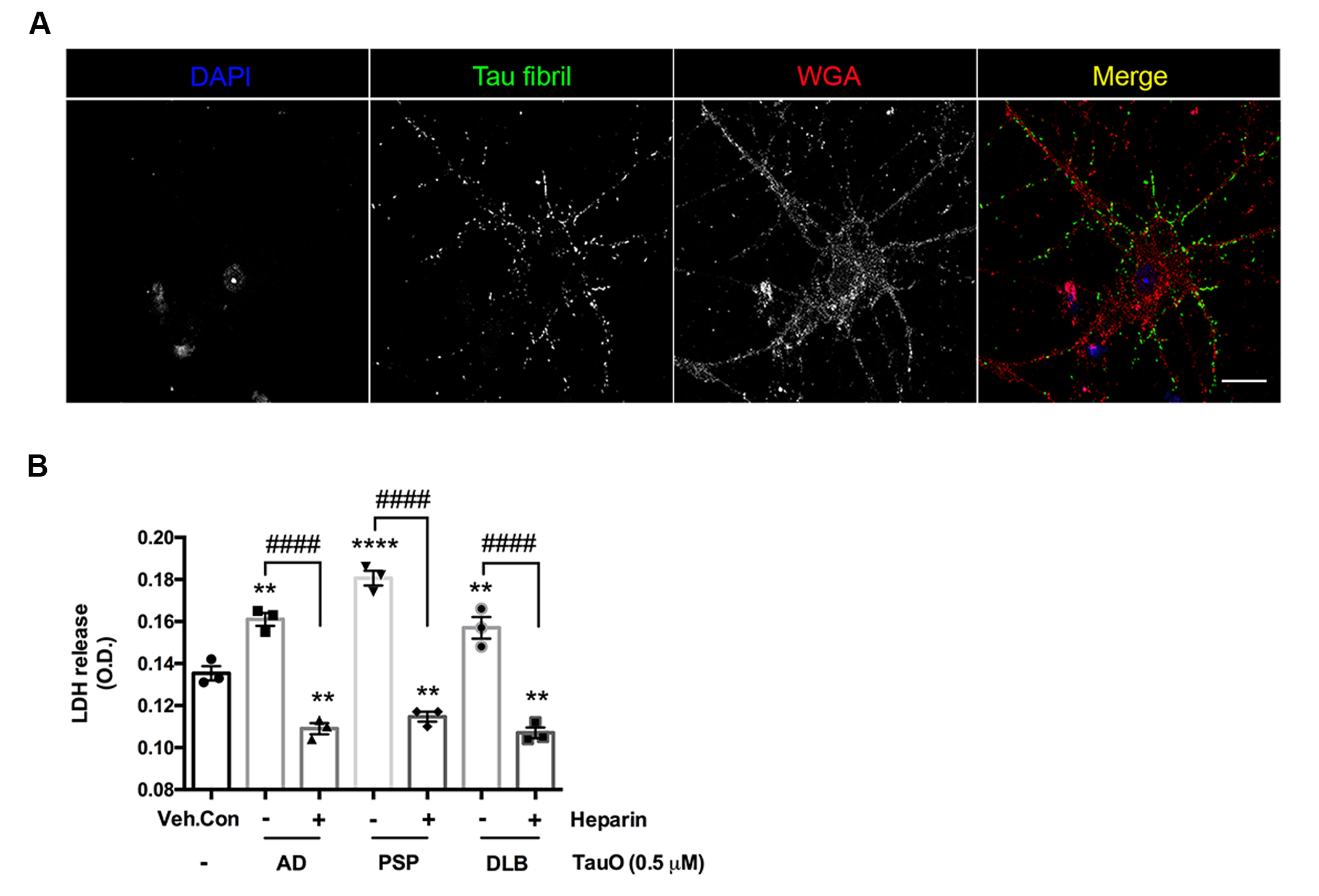

Supplement: Supplementary file 8 — Supplementary Figure S3 [file 41419_2020_2503_MOESM8_ESM.tif]

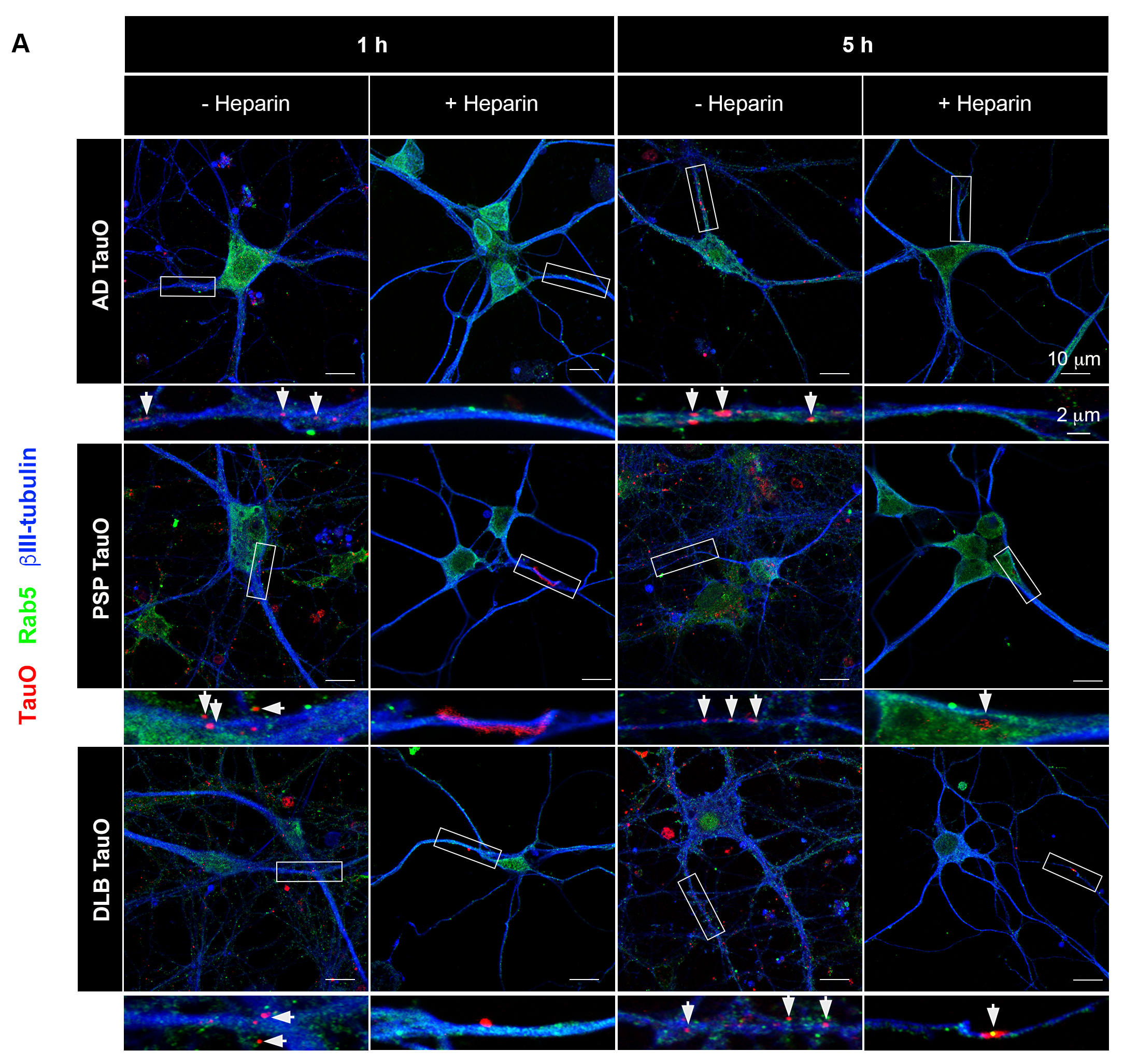

Supplement: Supplementary file 9 — Supplementary Figure S4 [file 41419_2020_2503_MOESM9_ESM.tif]

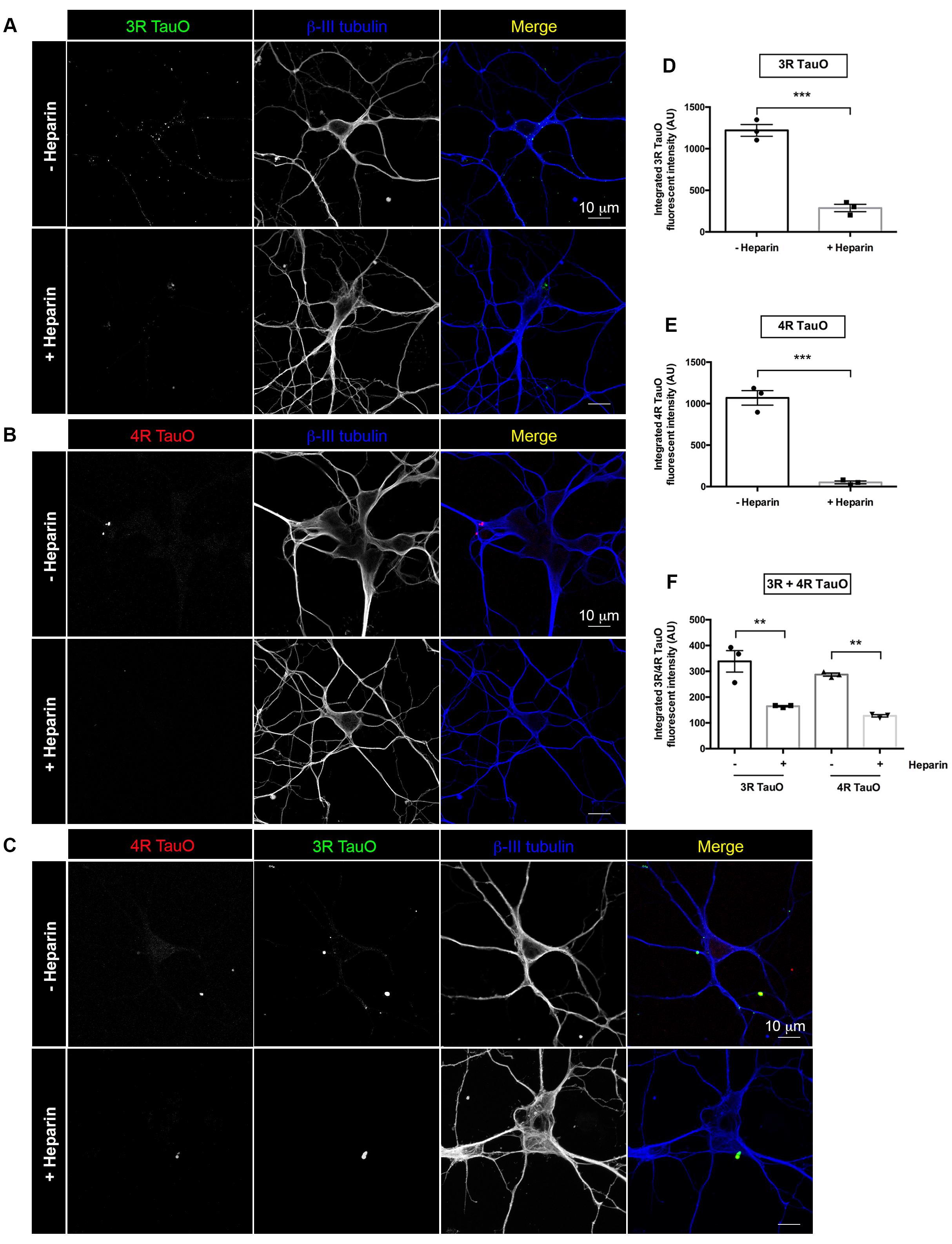

Supplement: Supplementary file 10 — Supplementary Figure S5 [file 41419_2020_2503_MOESM10_ESM.tif]

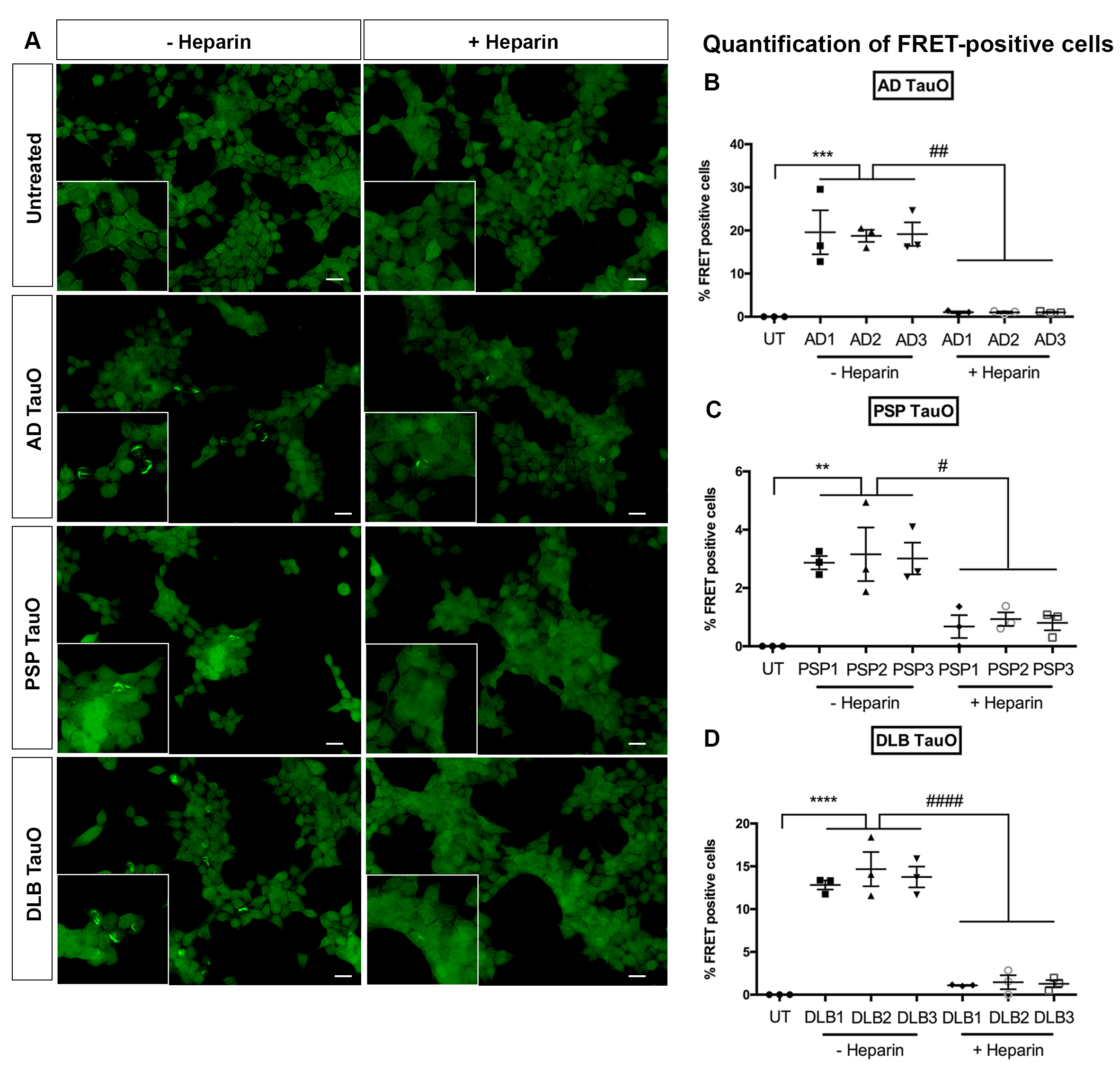

Supplement: Supplementary file 11 — Supplementary Figure S6 [file 41419_2020_2503_MOESM11_ESM.tif]
